# Multi-Scale Semantic Selection and Spatial Constraint-Guided Network for Cross-Scene Hyperspectral Image Classification

**DOI:** 10.3390/s26144627

**Published:** 2026-07-21

**Authors:** Yuntao Tang, Yu Sun, Xuyang Teng, Cuiping Yang, Ruifeng Xie, Xiaojun Guan, Xiaodong Yu

**Affiliations:** 1 College of Computer Science and Information Engineering, Harbin Normal University, Harbin 150025, China; 2024300691@stu.hrbnu.edu.cn (Y.T.); ycphsd@hrbnu.edu.cn (C.Y.); 2 Department of Municipal and Environmental Engineering, Harbin Vocational University of Architectural Technology, Harbin 150025, China; donglin_2016@nefu.edu.cn; 3 School of Communication Engineering, Hangzhou Dianzi University, Hangzhou 310018, China; tengxuyang@hdu.edu.cn; 4 College of Geography Science, Harbin Normal University, Harbin 150025, China; xieruifeng@hrbnu.edu.cn; 5 Teaching Experiment Equipment Management Center, Harbin Normal University, Harbin 150025, China

**Keywords:** hyperspectral image, cross-scene classification, domain generalization, multi-scale semantics, spatial constraint

## Abstract

Cross-scene classification of hyperspectral images attracts extensive research attention due to the prominent distribution discrepancies existing in hyperspectral images across different scenes. Most existing methods mitigate domain shift by expanding source domain samples and aligning feature distributions. However, these approaches fail to fully explore the spatial semantics of samples during the expansion process. Moreover, features are compressed into one-dimensional vectors in the alignment stage, resulting in the loss of critical spatial location information. To address the above issues, this paper proposes a multi-scale semantic selection and spatial constraint-guided network (MSCGnet). Specifically, the multi-scale semantic selection generator adopts a spatial diffusion scanning strategy to optimize the token serialization rule of Mamba. Pixels are arranged from the center to the periphery to maintain spatial continuity. Combined with the multi-scale semantic selection routing, multi-scale spectral–spatial features are extracted and a semantic selection matrix is constructed to guide Mamba to generate diverse augmented samples. The spatial constraint-guided discriminator leverages class activation map projection to impose explicit spatial constraints on feature distributions, further improving the reliability of augmented samples. Comprehensive experiments on multiple cross-scene HSI datasets demonstrate that the proposed method achieves superior classification accuracy and generalization performance with low model complexity.

## 1. Introduction

Hyperspectral Images (HSIs) collect ground object reflection information across hundreds of continuous spectral bands, which can finely characterize the physical and chemical properties of different ground objects. They have been widely applied in agricultural management [1], resource exploration [2], urban environmental monitoring [3], military reconnaissance [4] and other fields. In recent years, with the rapid development of deep learning, models based on Convolutional Neural Networks (CNNs) [5], Transformers [6], Mamba [7] and their hybrid architectures [8,9,10] have achieved remarkable progress in HSI classification. Nevertheless, these methods generally rely on large-scale labeled samples. Since HSI data labeling is complex and costly, the models suffer from insufficient generalization ability in practical applications [11]. To alleviate the limitations caused by scarce labeled samples, cross-scene HSI classification has become a popular research direction. Its goal is to utilize abundant labeled samples from the source domain to realize accurate classification on the target domain [12,13,14,15]. The main challenge lies in the significant distribution shift between domains. Differences in sensor types, acquisition time, illumination conditions and atmospheric environments across scenes lead to distinct data distributions, which drastically degrade model performance on the target domain [16].

To tackle domain shift, domain adaptation (DA) methods align feature distributions between the source and target domains to reduce cross-domain discrepancies [17,18,19,20]. According to differences in acting objects and feature levels, existing DA methods are divided into four categories: sample weight-based methods, statistical feature transformation-based methods, geometric feature transformation-based methods and adversarial learning-based methods. Sample weight-based methods assign differentiated weights to source domain samples to make the weighted source domain distribution approximate the target domain distribution and eliminate marginal distribution shift. Huang et al. [21] mined valid information using source domain labeled data and high-confidence pseudo-label samples from multiple target domains. They minimized the maximum mean discrepancy and covariance deviation between domains to align source and target domain distributions, and dynamically calculated weight coefficients based on domain distribution differences to fuse classification results, effectively improving cross-domain classification performance under single-source multi-target domain adaptation. Statistical feature transformation-based methods explicitly align statistical metrics such as mean, covariance and high-order moments of the source and target domains to reduce marginal or conditional distribution differences. Sun et al. [22] proposed the CORAL unsupervised domain adaptation algorithm, which reduced domain shift by aligning second-order statistics of two domains, and further extended it to deep CORAL for layer-wise matching in neural networks. Geometric feature transformation-based methods treat two domains as high-dimensional manifolds and realize spatial alignment via mapping, subspace projection and optimal transport. Hu et al. [23] presented a weighted domain adversarial neural network based on Wasserstein distance. A domain classifier was used to evaluate sample quality and assign corresponding weights, and high-quality cross-domain samples were adopted to optimize feature extraction and domain alignment. Zhang et al. [24] proposed discriminative cooperative alignment, which cooperatively aligns subspace geometry and marginal/conditional distributions under a data reconstruction constraint, effectively mitigating both geometric and statistical shift in cross-scene HSI classification. Adversarial learning-based methods adopt a minimax game: the feature extractor confuses the domain discriminator by mixing domain labels to learn domain-invariant features. Huang et al. [25] constructed an adversarial domain adaptation framework combining static convolution and dynamic instance convolution to extract coarse- and fine-grained features. A calibrated discriminator was used to align marginal domain distributions and revise pseudo-labels of the target domain. Combined with calibrated prototype loss, the method realized cross-domain category distribution alignment and boosted feature classification performance while preserving domain-invariant features. All the above DA algorithms require access to target domain data or prior information of the target domain during training, and can only optimize distribution alignment for known target domains [26].

In contrast, domain generalization (DG) methods do not rely on target domain data. They only use source domain information during training to enhance model generalization ability on unseen domains [27,28,29,30]. DG methods are categorized into data manipulation-based methods, representation learning-based methods and learning strategy-based methods according to different working levels. Data manipulation-based methods perform data transformation, augmentation and mixing on raw samples to expand the diversity of source domain data, so that the model can learn universal features from rich datasets. Peng et al. [31] proposed an adversarial domain augmentation algorithm to construct challenging virtual domain samples via adversarial training. Combined with a Wasserstein auto-encoder, the method relaxed worst-case constraints and introduced uncertainty quantification to effectively address the single-source DG problem. Representation learning-based methods impose constraints in the feature extraction stage to align feature distributions of multiple source domains, remove domain-specific interference information, and learn cross-domain universal and domain-invariant features, thereby eliminating inter-domain distribution differences in the feature space. Cheng et al. [32] took advantage of text prompts of vision foundation models and proposed a visual prompt tuning framework guided by text features. Large language models were used to disentangle text prompts and guide the model to extract domain-invariant visual features. Combined with domain-specific prototype learning to fuse unique information, the framework improved the cross-unseen-domain classification performance of DG models. Learning strategy-based methods do not modify data or feature alignment approaches. Instead, they optimize training paradigms and loss functions, and adopt training strategies such as meta-learning, multi-domain splitting, ensemble learning and regularization constraints to optimize parameter searching directions and enhance model generalization on unseen domains. Li et al. [33] designed a DG training paradigm based on meta-learning. Virtual test domains were constructed within mini-batches to simulate domain shift, and meta-optimization constraints were applied to synchronously optimize the training process and improve the accuracy on virtual test domains, so as to strengthen model generalization on unseen domains.

Although the above methods have achieved great success in natural image tasks, they still have limitations when applied to HSIs. HSIs possess complex spectral–spatial coupling characteristics. Existing methods usually ignore the diversity of multi-scale spatial semantic information inside samples during feature transformation, and adopt a unified transformation for features at different spatial positions. As a result, the generated augmented samples lack diversity in spatial semantics and cannot fully cover the feature distribution space of the target domain. In addition, most existing methods compress feature maps into global one-dimensional vectors before imposing statistical constraints in feature distribution alignment. This dimensionality reduction operation discards the spatial location information of features, making it impossible for the alignment process to distinguish whether feature changes occur in semantically significant regions or background regions of HSIs. Consequently, the spatial constraints are coarse-grained and the supervision signals are inaccurate.

To solve the above problems, this paper proposes a multi-scale semantic selection and spatial constraint-guided network (MSCGnet). The proposed method jointly optimizes model generalization from two perspectives: modeling spatial semantic diversity in the sample expansion stage and maintaining spatial structure in the feature constraint stage. On the one hand, a multi-scale semantic selection generator (MSSG) is designed. A spatial diffusion scanning strategy (SDSS) is proposed to optimize the token serialization of Mamba and preserve local spatial neighborhood perception. Meanwhile, the multi-scale semantic selection routing (MSSR) extracts spectral–spatial features at different receptive field scales and generates a semantic selection matrix, which guides Mamba to dynamically learn transformation rules for different spatial semantic regions and produce augmented samples with rich spatial semantic differences. On the other hand, a spatial constraint-guided discriminator (SCGD) is developed. Class activation maps (CAMs) are used to construct spatial saliency constraints, extending feature distribution alignment from the traditional one-dimensional statistical space to the two-dimensional spatial structure space. This enables the model to focus on feature consistency in semantically critical regions and improves the quality of augmented samples and feature representation capability. The main contributions of this work are summarized as follows:

1. Different from existing Mamba-based HSI models, this work for the first time explicitly correlates Mamba’s token serialization rule with the unique center–edge spatial semantic prior of HSI patches. Rather than generic multi-directional scanning augmentation, SDSS acts as a dedicated sequencing mechanism designed for intrinsic HSI characteristics, in which patch central pixels dominate categorical core semantics.
2. Most existing domain generalization methods follow a two-stage pipeline: they first extract multi-scale features and then conduct feature-level alignment. In contrast, our proposed MSSR directly embeds the multi-scale semantic selection matrix into the internal state transition read/write matrices of the state space model (SSM), enabling semantic-aware modulation of SSM read and write operations. Such a design achieves tight intrinsic mechanism coupling, instead of simply attaching an independent multi-scale fusion module after feature extraction.
3. Current domain generalization methods either squeeze features into 1D vectors for global statistic alignment or leverage text priors to realize semantic alignment. By contrast, the proposed SCGD eliminates the demand for text priors entirely. It reuses weights from the classification head to generate class activation maps (CAMs), narrowing the constraint granularity from global statistics to pixel-level spatial activations. This effectively mitigates spatial position information degradation in the alignment process.
4. Extensive experiments on multiple public cross-scene HSI datasets verify that the proposed method outperforms state-of-the-art approaches while maintaining low parameter size and computational complexity, demonstrating its effectiveness and strong generalization ability.

## 2. Related Works

### 2.1. DG Methods for Cross-Scene HSI Classification

DG has become a crucial research direction for cross-scene HSI classification, as it can improve model generalization on unseen scenes without accessing target domain data. Different from DA methods, DG methods only utilize source domain data for training and enhance the adaptability to unseen target domains via data manipulation, representation learning and learning strategy optimization.

In data manipulation-based DG research for HSIs, Zhang et al. [34] proposed a Single-source Domain Expansion Network (SDEnet). It adopted dual encoders for semantics and morphology, combined with spatial and spectral randomization operations to generate expanded domain data. A discriminator with supervised contrast learning was used to aggregate features of homogeneous samples from the source and expanded domains, and adversarial training was applied to optimize the generator and enlarge feature distances between different categories. To mitigate inductive bias of styles in expanded data, Zhao et al. [35] put forward a Locally Linear Unbiased Randomization Network (LLURnet). Based on a symmetric encoder–decoder structure, it constructed local joint features via style randomization and realized data expansion using latent variables. The discriminator adopted intra-class and inter-class contrast regularization to build adversarial loss and balance domain-specific and domain-invariant features. Wang et al. [36] designed a Two-Stage Domain Alignment Single-source Domain Generalization Network (TSDAnet). An additional spectral learning branch was added to the generator to reduce the interference of noise on data generation. The discriminator adopted a pyramid feature fusion structure with dual projection heads, and a two-stage domain alignment strategy was used to alleviate domain shift. Gao et al. [37] proposed an Invariant Semantic Domain Generalization Shuffle Network (ISDGS). It expanded data content and styles while preserving domain-invariant semantics via feature style covariance, and a spatial shuffle discriminator was used to weaken the interference of domain-specific spatial structures on category semantics. Meanwhile, a dual-sampling adversarial contrast learning strategy was adopted to prevent the model from falling into local Nash equilibrium and improve model generalization on unseen scenes.

Representation learning-based DG methods focus on mining domain-invariant features and suppressing domain-related interference information. Qin et al. [38] proposed a Frequency Disentanglement and Data Geometry combined Domain Generalization Network (FDGnet). A frequency component separation mechanism was used to decouple domain-related and domain-irrelevant features, and data manifold geometric constraints were applied to maintain category structure consistency for robust cross-domain feature learning. Huang et al. [39] presented a Dynamic Token Augmented Mamba Network (DTAM). A dynamic token augmentation module was designed to perturb context information while preserving ground object semantics, generating diverse feature representations. Combined with random sample labels, a classification compensation loss was constructed to prevent excessive contraction of the feature space and improve model adaptability to unseen scenes. Chu et al. [40] proposed a Diversity-Driven Domain Generalization Network (RCRAnet). A diversity-guided regularization strategy was adopted at the data level to expand training distribution, and a rank-enhanced attention fusion mechanism was used to strengthen fine-grained feature extraction and cross-domain feature representation, alleviating feature homogenization in single-source training and boosting model performance on unseen scenes. In addition, some studies adopted causal inference to enhance domain generalization. Dong et al. [41] proposed a Spectral–Spatial Enhancement and Causal Constraint Network (S2ECnet). It jointly enhanced spectral and spatial feature representation and introduced causal constraints to suppress the interference of spurious correlation features on classification, enabling the model to focus on essential discriminative information for categories.

With the development of vision foundation models and multi-modal learning, linguistic information has been introduced into HSI DG tasks. Zhang et al. [42] proposed a Language-Aware Domain Generalization Network (LDGnet). Text semantic prompts were introduced to assist visual feature learning, and linguistic prior knowledge was used to enhance category semantic representation and guide the model to extract more stable domain-invariant features for better cross-scene classification performance. Wang et al. [43] designed an Explicit High-Level Semantic Network (EHSnet). High-level semantic representation was used to constrain feature distributions of samples from different domains, and category semantic consistency was enhanced to reduce inter-domain discrepancies and learn stable cross-domain discriminative features. Combined with the Mamba architecture, Jin et al. [44] proposed a Language-Guided Dual-Branch Mamba Network (LDBMamba). Local–global spatial scanning and finite-boundary spectral scanning were performed to extract spatial and spectral sequence features respectively, and a spectral–spatial star-shaped fusion module was used to suppress Mamba modules for efficient spectral–spatial feature modeling. Furthermore, a contrast learning strategy guided by label and text prior knowledge was introduced to enhance the learning of domain-invariant semantic representation and improve the generalization of cross-scene HSI classification.

Different from single-source DG methods that only use information from a single source domain, multi-source DG methods can mine richer domain-invariant features using multiple source domains. Qi et al. [45] proposed a Multi-source Domain Generalization Two-branch Network (MDGTnet), which consisted of a classifier, a domain-specific feature extraction branch and a domain-shared feature extraction branch. The former extracted imaging environment features unique to each source domain, while the latter captured cross-source domain universal features. Two-level feature fusion eliminated the interference of imaging environment and obtained highly discriminative category features. To further reduce model complexity and sensitivity to the number of labeled samples, Qi et al. [46] proposed a Shift Reduction Domain Generalization Network (SRDGnet). A spectral–spatial structure reconstruction strategy was adopted to jointly model local spectral features and global spectral–spatial features. A domain shift reduction module was designed to promote multi-scale feature interaction and inter-domain distribution alignment. Meanwhile, a lightweight feature extraction network and batch traversal constraint strategy were applied to improve sample utilization and training diversity, so as to learn more stable domain-invariant features.

### 2.2. State Space Models

In recent years, state space models (SSMs) derived from modern control theory have shown great potential in long-range sequence modeling. They combine the linear computational complexity O(L) of Recurrent Neural Networks (RNNs) and the parallel training advantage of Transformers, thus attracting widespread attention [47,48,49,50]. In particular, after the emergence of Mamba with the selective scan (S6) mechanism, vision-oriented SSM architectures have been rapidly applied to HSI processing tasks [51,52,53,54].

Mathematically, the traditional continuous-time SSM is a linear time-varying system [55]. It maps a one-dimensional input sequence x(t)∈R to an output sequence y(t)∈R via a latent state $h(t)\in R^{N}$. The control equations are formulated as follows:

$$h^{\prime }\left(t\right)=Ah\left(t\right)+Bx\left(t\right),$$

(1) y(t)=Ch(t)+Dx(t),

where $A\in R^{N\times N}$ denotes the state transition matrix, $B\in R^{N\times 1}$ is the input control matrix, $C\in R^{1\times N}$ represents the output projection matrix, D∈R is the direct transmission coefficient, and N stands for the state dimension.

Since the classic continuous SSM cannot be directly applied to discrete token processing in deep learning, Mamba adopts a Zero-Order Hold (ZOH) to discretize the continuous equations [56]. A data-adaptive time step parameter Δ∈R is used as the sampling interval. The discrete forms of the state transition matrix A and input control matrix B are given by the following:

$$\bar{A}=exp\left(\Delta A\right),$$

(2) $$\bar{B}={\left(\Delta A\right)}^{-1}\left(exp\left(\Delta A\right)-I\right)⊙\Delta B,$$

where I is the identity matrix and ⊙ denotes the Hadamard product. After discretization, the standard recursive form is written as follows:

$$h_{t}=\bar{A}h_{t-1}+\bar{B}x_{t},$$

(3) $$y_{t}=Ch_{t}+Dx_{t}.$$

On this basis, the selective scan (S6) mechanism of Mamba converts static discretization parameters into dynamic functions dependent on the input x [57], which greatly enhances the model’s ability to filter irrelevant information according to context. The adaptive parameterization is defined as follows:

$$B={Linear}_{B}\left(x\right),$$

$$C={Linear}_{C}\left(x\right),$$

(4) $$\Delta =Softplus\left(Parameter+{Linear}_{\Delta }\left(x\right)\right).$$

At present, many studies have explored the potential of Mamba in HSI classification. Li et al. [58] constructed a dual-branch structure with spatial Mamba and spectral Mamba to capture spatial structural dependencies and spectral correlations respectively, realizing collaborative modeling of global and local features and achieving a better trade-off between classification accuracy and efficiency. Zheng et al. [59] designed four complementary scanning paths including row-wise, column-wise, zig-zag and serpentine paths to realize multi-directional serialization modeling of 2D spatial structures. A multi-scale feature alternating mechanism was adopted to fuse fine-grained spectral information and hierarchical spatial semantics, enhancing the ability to model complex spectral–spatial dependencies. He et al. [7] transformed HSI data into 3D token sequences via a spectral–spatial token generation module, and designed a 3D spectral–spatial selective scan mechanism to perform selective state updates in both spectral and spatial dimensions for global spectral–spatial dependency modeling. Xu et al. [60] firstly introduced the mixture-of-experts mechanism into HSI classification. A Mamba mixture-of-experts module was constructed, and a sparse expert activation strategy was used to adaptively model different spectral–spatial patterns. Combined with an uncertainty-guided correction learning strategy, the model was guided to focus on regions with uncertain predictions and optimize feature representation, improving classification accuracy and robustness for complex scenes. Beyond HSI classification, direction-aware state space designs have also demonstrated strong potential in other remote-sensing restoration tasks. Sultan et al. [61] proposed a physics-guided four-directional state space network for remote-sensing image dehazing, combining linear-complexity axial scanning with partial differential equation-driven diffusion priors to achieve robust performance under nonuniform degradation with low model complexity.

Although existing Mamba-based methods have made progress in mining global spectral–spatial features of HSIs, they still have limitations when applied to cross-scene classification tasks. In standard Mamba architectures, the mapping matrices (B and C) responsible for writing and reading long short-term memory are completely derived adaptively based on local context within the source domain. They lack the ability to actively modulate and adapt to external unknown style perturbations such as macroscopic environmental changes and spectral shift across scenes. Consequently, the model tends to overfit the data manifold of the source domain, restricting its generalization performance on unseen scenes. Therefore, introducing explicit guidance of high-level semantics into SSMs to reconstruct robust domain-invariant features has become a critical problem to be solved.

## 3. Proposed Method

**Figure 1:**
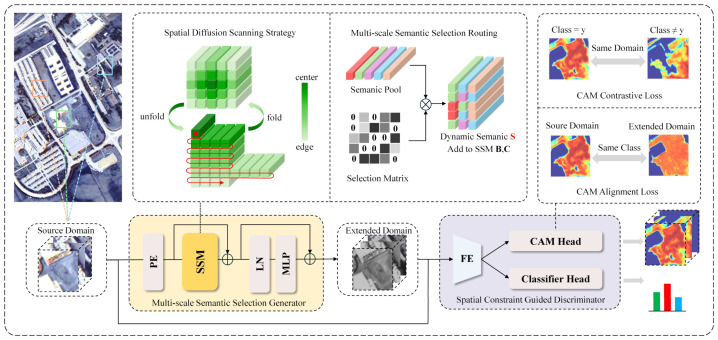
The workflow of the proposed MSCGnet. In the training phase, data augmentation is performed on source domain samples via a multi-scale semantic selection generator to generate extended domain samples with diverse feature distributions. Afterwards, both the source domain samples and extended domain samples are fed into the spatial constraint-guided discriminator, and the network is jointly optimized using classification loss, CAM alignment loss and CAM contrastive loss. In the testing phase, only the feature extraction backbone (FE) and classification head are retained to directly complete classification prediction on unseen target domain data.

The proposed MSCGnet consists of two core sub-modules: MSSG and SCGD. The overall framework is illustrated in Figure 1. Taking source domain HSI samples as input, MSSG generates reliable augmented samples with differentiated feature distributions relying on SDSS and MSSR. On the other hand, SCGD outputs classification predictions via the classification head and optimizes classification accuracy using cross-entropy loss. Meanwhile, class activation maps are derived inversely from classification weights. CAM alignment loss is adopted to ensure consistent spatial activation regions for the same category between source and extended domains, and CAM contrastive loss is used to enforce compact intra-class distribution and separated inter-class activation of CAMs. Explicit spatial constraints are imposed to suppress distortion of augmented samples and improve their effectiveness.

### 3.1. Multi-Scale Semantic Selection Generator

MSSG aims to adaptively mine multi-scale spatial semantic information of samples and guide the Mamba backbone to generate diverse extended domain samples. MSSG is composed of Position Encoding (PE), optimized selective scan module (SSM), Layer Normalization (LN) and Multi-Layer Perceptron (MLP) which contains two linear layers and one GELU layer. For input data, each sample is denoted as $X={\left\{x_{i}\right\}}_{i=1}^{B}\in R^{B\times C\times H\times W}$, where B is the batch size, C is the spectral dimension, and H×W represents the spatial size. The overall forward propagation of MSSG is formulated as follows:

$$x_{SSM}=SSM\left(PE\left(x\right)\right)+PE\left(x\right),$$

(5) $$x_{out}=MLP\left(LN\left(x_{SSM}\right)\right)+x_{SSM}.$$

The SSM integrates SDSS and MSSR: SDSS rearranges tokens in a center-to-periphery diffusion order to guarantee spatial continuity, while MSSR adaptively constructs a semantic selection matrix and generates semantic prompt vectors for each token. It adjusts state transition parameters in selective scans to guide the diverse generation of augmented samples. MLP further performs non-linear modeling and enhancement on features inside each token. With the collaboration of the above modules, MSSG can generate extended domain samples with more diverse feature distributions while maintaining the integrity of sample spatial semantics.

#### 3.1.1. Spatial Diffusion Scanning Strategy

Standard Mamba unfolds 2D feature maps into 1D sequences via sequential scanning. This scanning manner works well for natural language processing tasks, but it has obvious drawbacks when processing HSIs with distinct spatial structures. In patch-based cross-scene HSI classification, input patches are cropped around a central pixel to be classified. HSI ground objects present typical local spatial correlation: pixels closer to the geometric center share higher semantic consistency and spectral correlation with the central pixel, while peripheral pixels are more susceptible to mixed pixels, foreign object interference and boundary mutation. Traditional sequential scanning artificially enlarges the sequence distance between spatially adjacent pixels located in different rows after flattening 2D grids into 1D sequences, which completely destroys the original spatial continuity and fails to distinguish the contribution priority between central and peripheral pixels.

**Figure 2:**
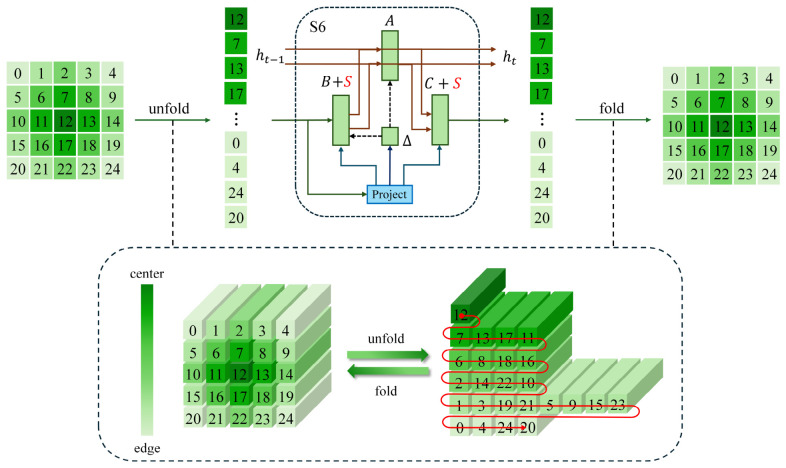
Flow chart of spatial diffusion scanning strategy.

To adapt to this characteristic of HSIs, we propose SDSS, as shown in Figure 2. The core idea of SDSS is to take the geometric center pixel of each patch as the origin and rearrange spatial tokens in an inward-to-outward annular diffusion order. For each spatial position (i,j) in an H×W feature map, the Euclidean distance from the patch center is calculated as follows:

(6) $$d\left(i,j\right)=\sqrt{{\left(i-\frac{H-1}{2}\right)}^{2}+{\left(j-\frac{W-1}{2}\right)}^{2}}.$$

The definition of the central coordinate in Equation (6) is only valid for square image patches where H=W. For rectangular patches with H≠W, the original Euclidean distance introduces anisotropic bias, such that edge pixels along the longer side are misjudged as closer to the center. We therefore propose the anisotropic distance formula as follows:

(7) $$d(i,j)=\sqrt{{\left(\frac{i-\frac{H-1}{2}}{H}\right)}^{2}+{\left(\frac{j-\frac{W-1}{2}}{W}\right)}^{2}},$$

Equation (7) performs dimensional normalization on the horizontal and vertical coordinate offsets separately to eliminate anisotropic bias induced by non-square image patches. It preserves the semantic sorting principle of center priority, periphery deferral under arbitrary aspect ratios. For irregular non-rectangular regions such as superpixel segments, the Euclidean distance adopted in SDSS can be replaced with the shortest geodesic distance to the central pixel calculated on the regional adjacency graph, which further extends the applicable scope of the proposed strategy.

The sorting strategy based solely on Euclidean distance implies the assumption that pixels closer to the central pixel possess higher semantic relevance. To address this issue, we extend the geometric distance to a spatial semantic distance formulated as follows:

(8) $$d_{sem}(i,j)=d(i,j)\cdot exp(1-cos(x_{c},x_{i,j})),$$

where d(i,j) denotes the original Euclidean distance, and $cos(x_{c},x_{i,j})$ represents the cosine similarity between the spectral vectors of the central pixel and the target pixel. For pixels with low spectral similarity to the central pixel, the exponential term rises, pushing such pixels backward in the sorting order. This allows the Mamba model to first model core regions with strong semantic consistency and then gradually incorporate ambiguous boundary areas, overcoming the drawback that simple geometric distance fails to distinguish land cover boundaries.

All N=H×W spatial positions are sorted in ascending order of distance to obtain a spatial diffusion sorting index sequence:

(9) $$I=argsort\left(\{d\left(i,j\right){\}}_{i,j}\right),$$

the input feature $x\in R^{B\times N\times C}$ is reordered using I to generate a feature sequence arranged in center-to-periphery diffusion order:

(10) $$x_{sort}=unfold\left(x,I\right),$$

the reordered sequence $x_{sort}$ is fed into the Mamba selective scan module for semantics-guided sequence modeling to produce $y_{sort}$. After sequence processing, the inverse sorting index $I^{-1}$ is used to restore the output features to the original spatial layout:

(11) $$y=fold\left(y_{sort},I^{-1}\right).$$

#### 3.1.2. Multi-Scale Semantic Selection Routing

**Figure 3:**
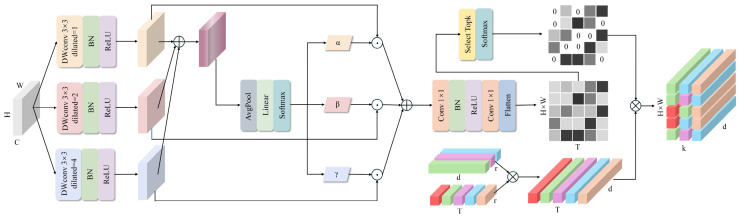
Flow chart of multi-scale semantic selection routing.

In cross-scene HSI classification, spatial contexts at different scales complement each other in describing sample semantics. Small receptive fields capture fine-grained spatial details such as local edges and textures, while large receptive fields model global regional structures and ground object distribution patterns. Feature extraction based on a single scale leads to one-sided semantic representation and limits the diversity of generated augmented samples. To enable the Mamba backbone to fully utilize multi-scale spatial semantics when generating augmented samples, we design MSSR, as illustrated in Figure 3. It adaptively fuses multi-scale features to construct a sparse semantic selection matrix, generates personalized semantic prompt vectors for each spatial token, and dynamically modulates SSM parameters of Mamba.

Given a spatial feature $x\in R^{B\times C\times H\times W}$, MSSR first extracts multi-scale spatial features via three parallel dilated depthwise separable convolution branches with dilation rates of 1, 2 and 4, corresponding to equivalent receptive fields of 3 × 3, 5 × 5 and 9 × 9 respectively, covering spatial scales from local to global:

(12) $$f_{r}=ReLU\left(BN\left({DWConv}_{3\times 3,r}\left(x\right)\right)\right),$$

where dilation rate r∈{1,2,4}. Features from three branches are summed element-wise to obtain the initial multi-scale fused feature, and global average pooling is applied to compress spatial dimensions to generate channel-wise descriptors:

(13) $$S=AvgPool\left(\sum_{r\in \{1,2,4\}}f_{r}\right).$$

Inspired by SKNet [62], a channel attention mechanism is adopted to adaptively estimate the contribution weight of each scale branch for different channels:

(14) $$\left[\alpha ,\beta ,\gamma \right]=Softmax\left(\left[{FC}_{1}\left(S\right),{FC}_{2}\left(S\right),{FC}_{3}\left(S\right)\right]\right),$$

where α, β and γ are adaptive channel weights corresponding to dilation rates 1, 2 and 4. Three branches are weighted and fused to obtain the adaptive multi-scale aggregated feature:

(15) $$F=\alpha ⊙f_{1}+\beta ⊙f_{2}+\gamma ⊙f_{4},$$

where ⊙ denotes element-wise multiplication. With this adaptive weighting mechanism, MSSR dynamically adjusts the contribution of each scale branch according to the feature distribution of different samples and channels, realizing content-adaptive scale selection instead of simple equal-weight superposition in multi-scale feature fusion.

After obtaining the multi-scale aggregated feature F, a lightweight selection score projection head is used to map F into a semantic selection score matrix P. The number of channels is gradually compressed from C to a preset number of semantic prototypes T. Then spatial dimensions are flattened and transposed to obtain $M\in R^{B\times N\times T}$, where N=H×W is the total length of spatial token sequences. Each row of M represents the matching score of the multi-scale semantic feature of each spatial token with T semantic prototypes.

Next, top-k selection is performed on each token of M to retain only the indices of the top-k semantic prototypes with the highest scores. Softmax is applied to the corresponding top-k scores to generate sparse non-zero weights, while weights of unselected prototypes are set to zero. The final spatial semantic selection matrix is derived via sparse scattering:

(16) P=Scatter(Softmax(Topk(M,k))).

The top-k sparse selection mechanism has two main functions. First, sparsity constraints enable tokens at different spatial positions to activate different combinations of semantic prototypes, providing spatially differentiated semantic guidance for SSM modulation of Mamba. Second, sparse selection avoids over-smoothing caused by full activation of all prototypes in fully connected paths, leading to clearer and more deterministic semantic selection and facilitating the generation of augmented samples with distinct semantic distributions.

To theoretically verify that this top-k strategy inherently restrains redundant correlations between semantic prototypes, we supplement the rigorous mathematical derivation of the matrix construction as follows. Let $M\in R^{N\times T}$ denote the original matching score matrix. For the row vector $m_{i}$ corresponding to the i-th token, the top-k sparse filtering operator is defined as follows:

(17) $$P_{i,j}=\{\begin{array}{cc} \frac{\exp(m_{i,j})}{\sum_{j^{\prime }\in {id}_{k}(m_{i})}\;\exp(m_{i,j^{\prime }})}, & \;j\in {id}_{k}(m_{i})\\ 0, & \;\mathrm{otherwise} \end{array},$$

it can be observed from this definition that each row of P contains at most k non-zero entries and satisfies $\sum_{j}P_{i,j}=1$. Based on this, we derive that the joint activation probability of any two distinct semantic prototypes $e_{p},e_{q}$ (p≠q) across all tokens satisfies the following:

(18) $$Pr(p,q\in {idx}_{k}(m_{i}))\leq \frac{k(k-1)}{T(T-1)},$$

when k≪T, the probability that two arbitrary prototypes are simultaneously selected by the same token is explicitly suppressed to the order of $O\left(k^{2}/T^{2}\right)$, which demonstrates that the top-k mechanism itself imposes implicit constraints on excessive correlation among semantic prototypes.

A low-rank decomposition strategy is adopted to construct interpretable and learnable semantic pools, which consist of a low-rank semantic embedding dictionary $E\in R^{T\times r}$ and a state space projection matrix $T\in R^{r\times d}$, where r is the low-rank dimension and d is the state dimension of Mamba. The low-rank decomposition maintains the representation capability of semantic embeddings while controlling the parameter scale. The semantic selection matrix P is used to weight and aggregate semantic prototype embeddings to generate dynamic semantics for each spatial token:

(19) S=P⋅(E⋅T).

Since each row of P only contains k non-zero elements, the semantic prompt vector of each position in S is actually a sparse weighted combination of k semantic prototype embeddings. Tokens at different spatial positions obtain differentiated semantic prompts by activating different prototype combinations. Such spatial differentiation is the fundamental mechanism driving Mamba to generate augmented features with diverse distributions.

Furthermore, to quantify the amount of original multi-scale semantic information lost during low-rank approximation, we derive the error bound for low-rank semantic pooling decomposition in the following part. Let $W^{*}\in R^{T\times d}$ denote the full-rank semantic prototype embedding matrix. Ideally, each semantic prototype holds an independent d-dimensional semantic representation. Instead of directly optimizing the full-rank matrix, we practically learn its low-rank approximation $\hat{W}=ET$ subject to the rank constraint $\mathrm{rank}\left(\hat{W}\right)\leq r$. According to the Eckart–Young–Mirsky theorem, the lower bound of the optimal rank-r approximation error measured by the Frobenius norm is formulated as follows:

(20) $$∥W^{*}-\hat{W}{∥}_{F}\geq \sqrt{\sum_{i=r+1}^{min\left(T,d\right)}\sigma_{i}^{2}\left(W^{*}\right)},$$

where $\sigma_{i}\left(W^{*}\right)$ refers to the i-th largest singular value of $W^{*}$. This inequality quantitatively describes the minimal inevitable semantic information loss caused by compressing full-rank semantic embeddings into a low-rank space of dimension r.

#### 3.1.3. Mamba State Space Modulation Based on Dynamic Semantics S

Inspired by [63], SSMs can be mathematically analogous to linear attention mechanisms. Given query matrix Q, key matrix K and value matrix V, the formula of linear attention is written as follows:

(21) $$y_{i}=\frac{Q_{i}\left(\sum_{j=1}^{i}K_{j}^{⊤}V_{j}\right)}{Q_{i}\left(\sum_{l=1}^{i}K_{l}^{⊤}\right)},$$

let $S_{i}=\sum_{j=1}^{i}K_{j}^{⊤}V_{j}$ and $Z_{i}=\sum_{l=1}^{i}K_{l}^{⊤}$; then Equation (21) is rewritten as follows:

$$S_{i}=IS_{i-1}+K_{i}^{⊤}V_{i},$$

(22) $$y_{i}=Q_{i}S_{i}/Q_{i}Z_{i}+Ox_{i},$$

where I and O denote the identity matrix and zero matrix respectively, and $x_{i}$ is the input token at step i. According to the state space equation in Equation (3) and the approximation $\bar{B}x_{i}\approx \Delta Bx_{i}=B\left(\Delta x_{i}\right)$, Equation (3) is transformed into the following general form:

$$h_{i}={\bar{A}h}_{i-1}+B\left(\Delta x_{i}\right),$$

(23) $$y_{i}=Ch_{i}/I+Dx_{i}.$$

By comparing Equations (22) and (23), we obtain the corresponding relationships between SSMs and linear attention: $h_{i}∼S_{i}$, $B∼K^{⊤}$, C∼Q. In attention mechanisms, the key matrix K determines how input information is written into the memory state $S_{i}$, and the query matrix Q controls how information is read from the memory state. Correspondingly, in SSMs, B regulates the updating intensity of the hidden state $h_{i}$ by current input $x_{i}$, and C governs the projection from hidden state to output. Therefore, jointly modulating B and C is semantically equivalent to modulating keys and queries in attention mechanisms simultaneously, i.e., intervening both memory writing and reading processes. This enables SSMs to accumulate and output information adaptively according to semantic characteristics of current positions during sequence propagation.

Based on the above analogy, the dynamic semantic term S is added as an additive modulation term to both the input control matrix B and output projection matrix C. The derivation of modulated discrete state space is as follows:

$$h_{t}=\bar{A}h_{t-1}+\bar{\left(B+S\right)}x_{t},$$

(24) $$y_{t}=(C+S)h_{t}+Dx_{t},$$

where $\bar{\left(B+S\right)}={\left(\Delta A\right)}^{-1}\left(exp\left(\Delta A\right)-I\right)⊙\Delta \left(B+S\right)$. The dynamic semantics S carry multi-scale semantic prototype information adaptively selected by each token via MSSR. Injecting S into **B** introduces semantic priors into the key matrix, so that the way input information updates the hidden state is explicitly guided by semantic characteristics of current positions. Channels with strong activation of semantic prototypes enable input information to update hidden states more effectively and accumulate richer semantically relevant information. Injecting S into **C** guides the model to selectively extract information related to current position semantics from hidden states, so that output features can accurately reflect the semantic attribution of current tokens rather than relying on a general projection manner.

To theoretically verify the reliability of the model recursion after introducing the semantic modulation term, we further analyze the impact of the semantic modulation term on the stability of state recursion and provide a complete theoretical proof of convergence, with derivations presented as follows.

For Equation (24), the stability of the system is determined by the spectral radius of the state transition matrix $\bar{A}=exp(\Delta A).$ The additive semantic modulation matrix S at the input side is integrated into the input coefficient $\bar{\left(B+S\right)}$, acting only on the input $x_{t}$ without altering the homogeneous state transition term $\bar{A}h_{t-1}.$ The original Mamba adopts HiPPO initialization via diagonalization with negative real parts to guarantee $∥\bar{A}∥=\rho <1.$ Unfolding the recursive relation term by term yields the following:

(25) $$h_{t}={\bar{A}}^{t}h_{0}+\sum_{k=1}^{t}{\bar{A}}^{t-k}\bar{\left(B+S\right)}x_{k},$$

taking the norm of both sides and applying the triangle inequality ∥U+V∥≤∥U∥+∥V∥ as well as the submultiplicativity of operator norms ∥MN∥≤∥M∥∥N∥ for bounding:

(26) $$∥h_{t}∥\leq \rho^{t}∥h_{0}∥+\left(∥\bar{B}∥+∥\bar{S}∥\right)∥x{∥}_{\infty }\sum_{i=0}^{t-1}\rho^{i},$$

evaluating the finite geometric series sum $\sum_{i=0}^{t-1}\rho^{i}=\frac{1-\rho^{t}}{1-\rho }$ and substituting $\rho =∥\bar{A}∥$, we obtain the upper bound of the hidden state:

(27) $$∥h_{t}∥\leq ∥\bar{A}{∥}^{t}∥h_{0}∥+\frac{1-∥\bar{A}{∥}^{t}}{1-∥\bar{A}∥}\left(∥\bar{B}∥+S_{max}\right)∥x{∥}_{\infty },$$

after Softmax normalization in MSSR, Equation (19) inherently satisfies $∥\bar{S}{∥}_{2}\leq ∥ET{∥}_{2}=S_{max}$, which ensures the norm of the modulation matrix is bounded. As t→∞, $∥\bar{A}{∥}^{t}\to 0$, and the hidden state converges to the steady-state upper bound $\frac{\left(∥\bar{B}∥+S_{max}\right)∥x{∥}_{\infty }}{1-∥\bar{A}∥}.$ No state divergence will be induced by the introduction of the semantic modulation term.

### 3.2. Spatial Constraint-Guided Discriminator

Existing feature distribution alignment methods usually compress feature maps into global one-dimensional vectors before minimizing statistical distances. This operation discards spatial location information of features, making it impossible for the alignment process to distinguish whether feature changes occur in target object regions or background regions, which may lead to invalid or even harmful feature alignment.

**Figure 4:**
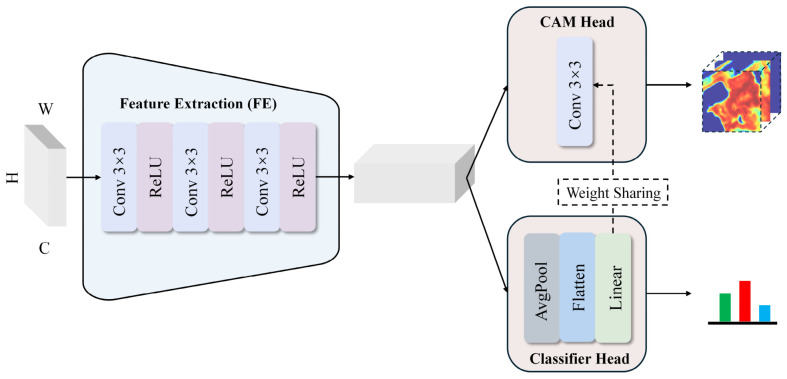
Flow chart of spatial constraint-guided discriminator.

Inspired by [64], we introduce CAM projection into the discriminator to refine feature distribution constraints from the global statistical level to the spatial activation location level, realizing fine-grained supervision for the semantic reliability of augmented samples. The structure of SCGD is shown in Figure 4. It consists of a feature extraction (FE) backbone stacked by three cascaded 3 × 3 convolution and ReLU activation blocks, a classification head composed of global average pooling, flattening layer and linear layer, and a CAM head which reuses weights of the linear layer in the classification head for spatial activation projection via weight sharing. The overall forward propagation of SCGD is formulated as follows:

F=FE(x),

p=Linear(Flatten(AvgPool(F))),

(28) $${CAM}_{c}\left(x,y\right)=\sum_{k=1}^{K}w_{c,k}\cdot F_{k}\left(x,y\right)+b_{c},$$

where F denotes the feature map output by the feature extraction backbone, p is the category prediction probability, ${CAM}_{c}\left(x,y\right)$ represents the activation value of the c-th category at spatial position (x,y), $F_{k}\left(x,y\right)$ is the response value of the k-th feature channel at position (x,y), $w_{c,k}$ is the weight corresponding to the k-th feature channel for the c-th category in the linear layer of the classification head, and $b_{c}$ is the bias term of the c-th category.

During training, SCGD processes both source domain samples and extended domain samples and imposes supervision constraints from three aspects: cross-entropy classification loss ensures basic discriminative capability, CAM alignment loss constrains the consistency of spatial activation locations, and CAM contrastive loss enhances the discriminability of category activation. The three components jointly form the complete training objective of SCGD.

Cross-entropy loss is adopted as the classification loss. SCGD performs classification prediction on source domain samples $\{x_{i},y_{i}{\}}_{i=1}^{B}$ and extended domain samples $\{\bar{x_{i}}{\}}_{i=1}^{B}$ generated by the generator, and the joint cross-entropy classification loss is calculated as follows:

(29) $$L_{ce}=-\frac{1}{B}\sum_{i=1}^{B}\sum_{c=1}^{C}y_{i}^{c}\left(logp_{i}^{c}+log{\bar{p}}_{i}^{c}\right),$$

where $y_{i}^{c}$ is the true label of the i-th sample for the c-th category, $p_{i}^{c}$ and ${\bar{p}}_{i}^{c}$ denote the predicted probability of the c-th category for source domain samples and extended domain samples respectively, and C is the total number of categories. Since extended domain samples are transformed from source domain samples and inherit the same category labels, they can be supervised uniformly in one loss term. $L_{ce}$ guarantees that the model maintains stable category discriminability in the expanded feature space and lays a foundation for subsequent spatial constraints.

On the basis of classification supervision, further spatial constraints are introduced. Let ${CAM}_{c}\left(x,y\right)$ be the class activation map of source domain sample x processed by SCGD, and ${\bar{CAM}}_{c}\left(x,y\right)$ be the corresponding class activation map of extended domain sample $\bar{x}$. The CAM alignment loss is defined as the mean square error between the two CAMs across all categories and spatial positions:

(30) $$L_{align}=\frac{1}{CHW}\sum_{c=1}^{C}\sum_{x=1}^{H}\sum_{y=1}^{W}{\left({\bar{CAM}}_{c}\left(x,y\right)-{CAM}_{c}\left(x,y\right)\right)}^{2},$$

Equation (30) assigns an identical weight to every spatial position, without distinguishing whether the pixel belongs to a semantically critical foreground region or an irrelevant background region. A foreground-aware weight $\omega_{c}(x,y)$ can be introduced to adaptively strengthen the constraint on highly activated regions while retaining a baseline weight for background regions. The refined CAM alignment loss is thus reformulated as follows:

(31) $$L_{align}=\frac{1}{CHW}\sum_{c=1}^{C}\sum_{h=1}^{H}\sum_{w=1}^{W}\omega_{c}\left(x,y\right)\cdot {\left({CAM}_{c}\left(x,y\right)-{\bar{CAM}}_{c}\left(x,y\right)\right)}^{2},$$

where the foreground weight $\omega_{c}\left(x,y\right)=1+1\left[{CAM}_{c}\left(x,y\right)>{\bar{\theta }}_{c}\right]$, ${\bar{\theta }}_{c}$ refers to the adaptive threshold of source domain CAM for class c which equals the mean CAM response of this class plus one standard deviation, and 1[⋅] is the indicator function. With this design, highly activated foreground regions are assigned larger weights in the alignment loss, whereas the weights of background regions remain 1. Therefore, the spatial weight distribution of the loss function conforms to the distribution property of land features that targets are concentrated while backgrounds are sparse.

CAM contrastive loss $L_{con}$ is designed to strengthen spatial constraints from the perspective of statistical characteristics of category activation. It consists of intra-class compactness loss $L_{intra}$ and inter-class separation loss $L_{inter}$. For category c, the spatial mean $\mu_{c}$ and spatial variance $\sigma_{c}^{2}$ of its CAM are calculated first:

$$\mu_{c}=\frac{1}{HW}\sum_{x=1}^{H}\sum_{y=1}^{W}{CAM}_{c}\left(x,y\right),$$

(32) $$\sigma_{c}^{2}=\frac{1}{HW}\sum_{x=1}^{H}\sum_{y=1}^{W}{\left({CAM}_{c}\left(x,y\right)-\mu_{c}\right)}^{2},$$

the intra-class compactness loss is defined as the average spatial variance of CAMs of source domain samples and extended domain samples for the true category y:

(33) $$L_{intra}=\frac{1}{2}\left(\sigma_{y}^{2}+{\bar{\sigma }}_{y}^{2}\right),$$

where $\sigma_{y}^{2}$ and ${\bar{\sigma }}_{y}^{2}$ are the CAM spatial variances of source domain samples and extended domain samples for the true category y respectively. Minimizing $L_{intra}$ encourages the model to concentrate attention for the target category on spatially consistent regions and avoid unstable localization caused by scattered activation. To enlarge the gap of CAM mean values between the true category and the most confusing category, the most confusing category $c^{*}$ with the maximum CAM spatial mean among categories excluding the true category is identified first:

(34) $$c^{*}=arg\underset{c\neq y}{max}\mu_{c},$$

then a hinge loss is adopted to construct the inter-class separation loss with a margin parameter γ:

(35) $$L_{inter}=max\left(0,\;\gamma -\left|\mu_{y}-\mu_{c^{*}}\right|\right),$$

when the difference in CAM spatial means between the true category y and the most confusing category $c^{*}$ is smaller than γ, the loss is positive and prompts the optimization process to further enlarge the activation gap. When the gap exceeds γ, the loss equals zero and no additional constraints are applied, so that computing resources are focused on challenging category boundaries. The final contrastive loss is the sum of intra-class loss and inter-class loss:

(36) $$L_{con}=L_{intra}+L_{inter}.$$

Combining the above three loss terms, the overall training objective of MSCGnet is defined as follows:

(37) $$L_{total}=L_{ce}+\lambda_{1}L_{con}+\lambda_{2}L_{align},$$

where $\lambda_{1}$ and $\lambda_{2}$ are hyperparameters to balance the contribution of each loss term. The three loss terms form a progressive constraint system from coarse to fine: $L_{ce}$ guarantees basic discriminability at the category level, $L_{align}$ constrains the consistency of augmented samples and source domain samples at the spatial location level, and $L_{con}$ further strengthens intra-class compactness and inter-class separation at the statistical characteristic level. The three components work together to ensure the semantic reliability and feature diversity of augmented samples.

The training and testing process of MSCGnet is shown in Algorithm 1.
**Algorithm 1.** Pseudocode of MSCGnet1**Training stage:**2**Input:** Source domain samples $S=\{X_{i}^{s},Y_{i}{\}}_{i=1}^{N_{s}},$ total epoch number T.3**Output:** The parameters $\theta_{MSSG},\;$$\theta_{SCGD}$
4**Initialize:** $\theta_{MSSG},\;$$\theta_{SCGD}$5**For** epoch = 1: T **do:**6$\;\;x_{sort}=SDSS(X)$ through Equations (6) and (9)–(11)7  S=MSSR(X) through Equations (12)–(16) and (19)8$\;\bar{X}=MSSG(x_{sort},S)$ through Equations (5) and (24)9  **For all** X, $\bar{X}$:10  p,CAM=SCGD(X); $\bar{p},\bar{CAM}=SCGD(\bar{X})$ through Equation (28)11  Calculate the loss $L_{ce}$ through Equation (29)12  Calculate the loss $L_{align}$ through Equation (30)13  Calculate the loss $L_{con}$ through Equations (32)–(36)14  Calculate the total loss $L_{total}$ through Equation (37)15  **End For**16  Update $\theta_{MSSG},\;$$\theta_{SCGD}$ by gradient descent17**End For**18**Testing stage:**19**Input:** Target domain samples $T=\{X_{i}^{t},Y_{i}{\}}_{i=1}^{N_{t}}$
20**Load:** The parameters $\theta_{FE},\;$$\theta_{Classifier}$
21  F=FE(x); p=Classifier(F)22**Output:** p (Classification prediction)

## 4. Experiment and Discussion

To comprehensively verify the effectiveness and generalization ability of the proposed MSCGnet in cross-scene HSI classification, experiments are conducted on three public cross-scene HSI datasets: Houston, Pavia and YC. The Houston dataset reflects domain shift caused by different acquisition times, the Pavia dataset presents distribution discrepancies across different spatial scenes, and the YC dataset is used to evaluate cross-domain generalization under different sensor imaging conditions. Comprehensive evaluations on the three typical cross-scene tasks analyze the classification performance of MSCGnet and its capability to handle complex domain shift.

### 4.1. Experimental Datasets

**Figure 5:**
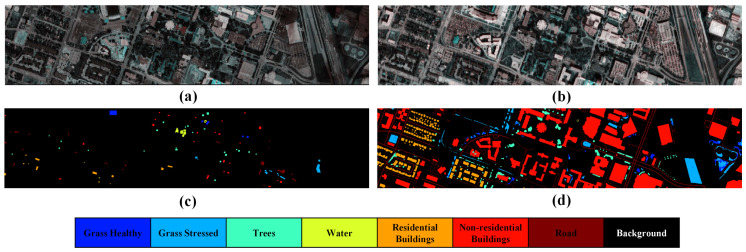
Pseudo-color image and ground-truth map of Houston dataset: (**a**) pseudo-color image of Houston 2013; (**b**) pseudo-color image of Houston 2018; (**c**) ground-truth map of Houston 2013; (**d**) ground-truth map of Houston 2018.

The Houston dataset contains two scenes: Houston 2013 [65] and Houston 2018 [66]. The two scenes are acquired by different sensors at different times over the campus and surrounding areas of the University of Houston. The Houston 2013 scene covers a spectral range of 380–1050 nm with 144 spectral bands, a spatial size of 349 × 1905 pixels and a spatial resolution of 2.5 m. The Houston 2018 scene shares the same wavelength range but only has 48 spectral bands with a spatial resolution of 1 m. The two scenes contain seven identical ground object categories. To meet experimental settings, 48 spectral bands matching Houston 2018 are selected from Houston 2013, and spatially overlapping regions of the two scenes are retained. Detailed category information and sample numbers are listed in Table 1. Pseudo-color images and ground-truth maps are shown in Figure 5. In this experiment, Houston 2013 is set as the source domain and Houston 2018 as the target domain.

**Figure 6:**
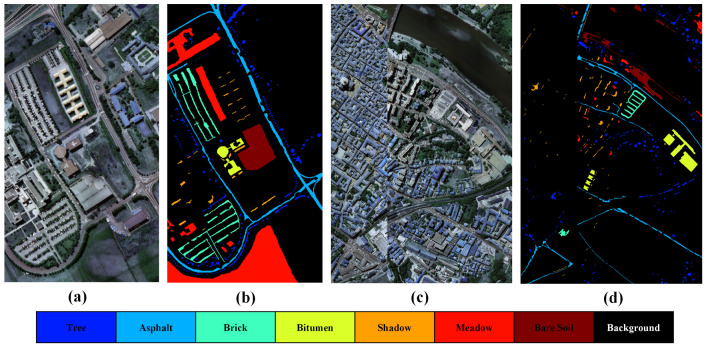
Pseudo-color image and ground-truth map of Pavia dataset: (**a**) pseudo-color image of University of Pavia; (**b**) ground-truth map of University of Pavia; (**c**) pseudo-color image of Pavia Center; (**d**) ground-truth map of Pavia Center.

The Pavia dataset includes two scenes: University of Pavia (UP) and Pavia Center (PC). Both scenes are collected by the ROSIS sensor in Pavia at different times [67]. The UP scene has 103 spectral bands ranging from 430 to 860 nm and a spatial size of 610 × 340 pixels. The PC scene contains 102 spectral bands covering the same wavelength range and a spatial size of 1096 × 715 pixels. The last band of the PC scene is removed to unify the spectral dimension with the UP scene. The two scenes share seven identical ground object categories. Detailed category information and sample numbers are presented in Table 2. Pseudo-color images and ground-truth maps are illustrated in Figure 6. In this experiment, University of Pavia is taken as the source domain and Pavia Center as the target domain.

**Figure 7:**
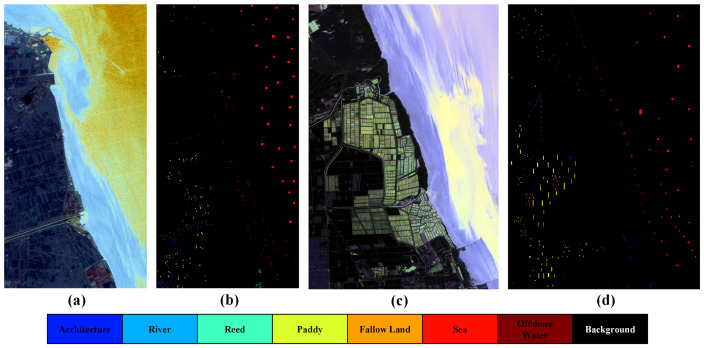
Pseudo-color image and ground-truth map of YC dataset: (**a**) pseudo-color image of GF-YC; (**b**) ground-truth map of GF-YC; (**c**) pseudo-color image of ZY-YC; (**d**) ground-truth map of ZY-YC.

The YC dataset consists of two scenes GF-YC and ZY-YC, acquired by AHSI sensors mounted on GF-5 and ZY1-02D satellites respectively. The datasets cover coastal wetland areas in Yancheng, Jiangsu Province, China, with spatial sizes of 1175 × 585 pixels and 1398 × 942 pixels respectively [68]. A total of 147 common spectral bands and seven shared ground object categories are selected for experiments. Detailed category information and sample numbers are shown in Table 3. Pseudo-color images and ground-truth maps are given in Figure 7. In this experiment, GF-YC is regarded as the source domain and ZY-YC as the target domain.

### 4.2. Experimental Setting

Three cross-scene HSI classification tasks are constructed, namely the Houston task, Pavia task and YC task. For all tasks, we partition labeled source domain samples into an 80% training subset and a 20% validation subset, where the validation set is exclusively derived from source domain data and utilized for hyperparameter tuning, model selection and early stopping. All hyperparameters are finalized according to performance on the source domain validation set without any access to target domain information. Target domain data are kept unseen throughout training and tuning; they are only used for one-shot final testing after the model architecture and all hyperparameters are fully fixed, and are never fed back into training, validation or parameter adjustment processes.

State-of-the-art DG algorithms are selected as comparison methods, including LDGnet [42], LLURnet [35], FDGnet [38], ISDGS [37], ADnet [69] and RCRAnet [40]. We also introduce two transfer learning methods originally developed for natural images, VREx [70] and GroupDRO [71], to conduct horizontal comparisons across different theoretical frameworks.

To ensure fair comparisons among different methods, all nine comparison methods (including the proposed MSCGnet) in this paper are trained and evaluated under unified experimental conditions. All models are trained from scratch based on the PyTorch 2.5.1 framework on an NVIDIA GeForce RTX 4060Ti GPU with 16 GB video memory. All methods share identical source training samples, and the source data are split into training and validation sets at a fixed ratio of 80%/20%. The unified preprocessing pipeline first applies global maximum normalization followed by pixel-wise L2 normalization. The Adam optimizer is uniformly adopted with a momentum of 0.9, an initial learning rate of 0.001, and an L2 weight decay of 0.0001. Fixed hyperparameters are set as a batch size of 256 and 200 training epochs. Overall Accuracy (OA) and Kappa Coefficient (KC) are used as uniform evaluation metrics. Ten repeated experiments with distinct random seeds are conducted consistently, and results are reported in the form of mean ± standard deviation. Only the patch size is not unified; each method adopts the optimal patch size recommended in its original paper to guarantee that every baseline operates under its optimal working condition.

For the Houston 2013 dataset with limited samples, two augmentation strategies, random flipping and random radiation noise, are uniformly utilized to expand the sample size to four times the original volume. Since LDGnet requires text prior information, coarse-grained and fine-grained text descriptions for the Houston and Pavia tasks follow the settings in the original literature. For the YC task which is not involved in existing works, coarse-grained and fine-grained semantic descriptions are designed for each category following the text construction principle of LDGnet and adopted uniformly in all related experiments to ensure fairness. Table 4 lists the fine-grained text descriptions for the YC task used in LDGnet.

### 4.3. Parameter Tuning

Sensitivity analysis is conducted on key hyperparameters of MSCGnet to explore their impacts on classification performance across three cross-scene tasks. The analyzed hyperparameters include patch size, semantic prototype count T, top-k sparsity coefficient k, loss weight coefficients $\lambda_{1}$ and $\lambda_{2}$. Candidate patch sizes are {9 × 9, 11 × 11, 13 × 13, 15 × 15, 17 × 17}, candidate values of T are {8, 16, 32, 64, 128}, candidate values of k are {1, 2, 3, 4, 5}, candidate values of $\lambda_{1}$ are {0.1, 0.25, 0.5, 0.75, 1}, and candidate values of $\lambda_{2}$ are {0.1, 0.5, 1, 5, 10}.

Table 5 presents OA values of MSCGnet with different patch sizes on three tasks. For the Houston and Pavia tasks dominated by artificial buildings, roads and trees in complex urban scenes, the patch size of 13 × 13 provides the most appropriate spatial context and achieves the highest OA (81.62% and 86.81% respectively). For the YC dataset with large-area contiguous natural ground objects such as reeds and offshore water, which have simple local textures and drastic boundary transitions, the optimal performance (86.47%) is obtained with a smaller patch size of 11 × 11. Larger patch sizes lead to decreased accuracy due to increased mixed pixels.

Table 6 reports the influence of the semantic prototype count T. When T increases from 8 to 32, the classification accuracy consistently improves on all three datasets because a larger prototype pool provides richer semantic representations for multi-scale semantic routing. However, further increasing T to 64 or 128 degrades performance. An excessively large prototype dictionary introduces redundant semantic prototypes that are rarely activated under the top-k routing mechanism, making semantic selection less discriminative and increasing optimization difficulty. Therefore, T=32 achieves the best trade-off between semantic diversity and prototype redundancy. Table 7 reports the classification results under three tasks with different top-k sparsity coefficient k. The highest cross-scene classification accuracy is achieved at k=3 for all tasks.

**Figure 8:**
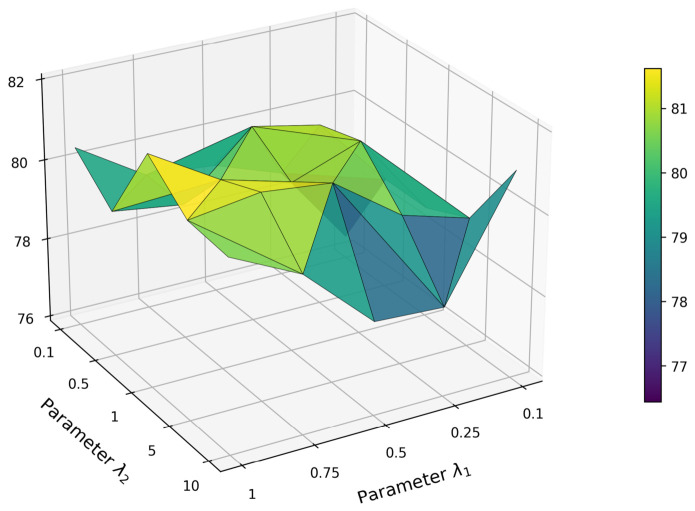
Parameter tuning of $\lambda_{1}$ and $\lambda_{2}$ on the Houston task.

**Figure 9:**
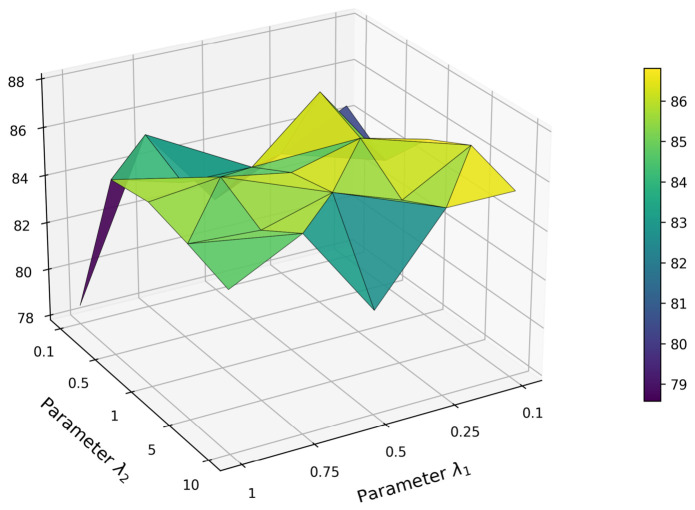
Parameter tuning of $\lambda_{1}$ and $\lambda_{2}$ on the Pavia task.

**Figure 10:**
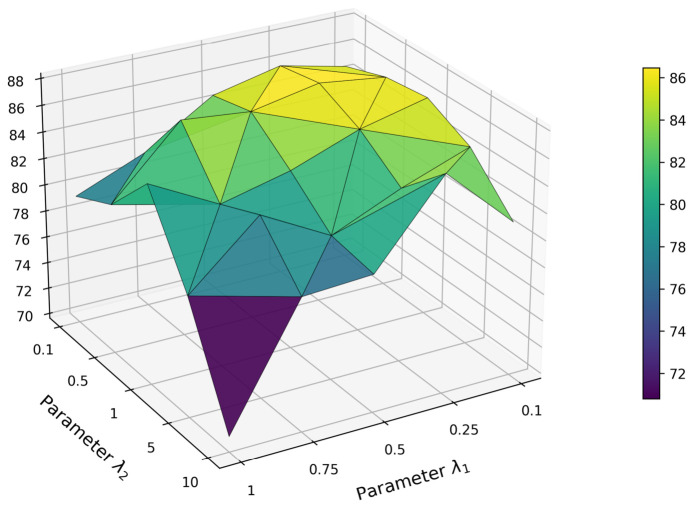
Parameter tuning of $\lambda_{1}$ and $\lambda_{2}$ on the YC task.

Figure 8, Figure 9 and Figure 10 show the joint parameter tuning results of $\lambda_{1}$ and $\lambda_{2}$ on three tasks. $\lambda_{1}$ controls the contribution of CAM contrastive loss $L_{con}$ to the total loss, and $\lambda_{2}$ controls the contribution of CAM alignment loss $L_{align}$. Both hyperparameters affect model performance, and the overall trends are stable, demonstrating good parameter robustness of MSCGnet. For the Houston task, the optimal performance is achieved when $\lambda_{1}=\lambda_{2}=1$, indicating that strong CAM alignment and contrast constraints help mitigate large cross-scene distribution discrepancies. For the Pavia and YC tasks, the optimal combination is $\lambda_{1}=0.25$ and $\lambda_{2}=0.5$. Moderate spatial constraints can effectively guarantee semantic consistency of augmented samples, while excessive constraints limit the diversity gain brought by expanded feature distributions. In general, reasonably balancing classification supervision and spatial constraint supervision can fully exploit the advantages of the proposed method and improve cross-scene generalization ability while ensuring semantic reliability of augmented samples.

We further conduct analytical derivation and quantitative verification for the optimal weights obtained via grid search from the theoretical perspective of gradient magnitude balancing. Let $∥\nabla_{\theta }L_{\alpha e}∥$, $∥\nabla_{\theta }L_{\alpha on}∥$ and $∥\nabla_{\theta }L_{align}∥$ denote the L2 gradient norms of the classification loss, contrastive loss and alignment loss with respect to the shared backbone parameters θ of the feature extractor, respectively. To ensure each loss term contributes at the same order of magnitude to parameter updates, the theoretically optimal weights satisfy the following equations:

$$\lambda_{1}\approx \frac{E\left[∥\nabla_{\theta }L_{ce}∥\right]}{E\left[∥\nabla_{\theta }L_{con}∥\right]},$$

(38) $$\lambda_{2}\approx \frac{E\left[∥\nabla_{\theta }L_{ce}∥\right]}{E\left[∥\nabla_{\theta }L_{align}∥\right]}.$$

We statistically sample the gradient norms of the three loss terms over the first 10 training epochs. A comparison between the theoretically calculated weight ratios and the empirical optimal values from grid search is presented in Table 8. As observed from the table, the theoretically derived weights and grid search optimal weights are of the same order of magnitude across all three datasets, with all relative deviations below 15%. This result fully verifies the validity of the proposed gradient magnitude balancing weighting strategy and provides a rigorous theoretical explanation for the optimal hyperparameters determined by grid search.

Analysis based on the sample distribution characteristics of each dataset reveals that the Houston dataset contains only 2530 labeled source domain samples. The scarcity of supervised samples inherently weakens the gradient magnitude of the classification loss. Accordingly, the optimal empirical weights are set to $\lambda_{1}=\lambda_{2}=1$ to compensate for insufficient classification supervision signals by increasing the weights of constraint losses. In contrast, the Pavia and Yicheng datasets possess abundant labeled source domain samples, and their classification loss gradients inherently contain sufficient discriminative information for category separation. Excessively large constraint weights would overcompress the feature space and suppress sample diversity. Thus, smaller weights $\lambda_{1}=0.25$ and $\lambda_{2}=0.50$ are adopted to impose regularization while retaining discriminative feature capacity.

### 4.4. Ablation Study

SDSS, MSSR, $L_{align}$ and $L_{con}$ are four key components of MSCGnet. Ablation experiments are carried out by removing each component one by one to evaluate their contributions to model performance. Quantitative results on three tasks are reported in Table 9.

The complete MSCGnet achieves the best performance on all three tasks, verifying the positive contribution of each component. Removing any component leads to performance degradation. Specifically, removing SDSS results in OA drops of 1.61%, 4.70% and 5.73% on three tasks respectively, which proves that SDSS plays a vital role in maintaining the model’s ability to perceive spatial continuity, and its removal directly degrades the overall understanding of HSI spatial semantics. Removing MSSR leads to OA reductions of 6.73%, 4.34% and 4.35%, which verifies the effectiveness of multi-scale semantic selection in guiding the generation of diverse samples. Removing $L_{con}$ causes OA decreases of 4.10%, 2.13% and 0.33%, indicating that CAM contrastive loss enhances the discriminability of category activation regions, promotes intra-class compactness and inter-class separation, and further improves the semantic distinguishability of augmented samples and cross-domain generalization of the model. Removing $L_{align}$ leads to the most significant performance drops, with OA decreasing by 7.94%, 10.88% and 4.51% respectively.

This demonstrates that CAM alignment loss is the core factor ensuring spatial semantic consistency of augmented samples. It constrains the spatial activation locations of augmented samples to be consistent with source domain samples, suppresses spatial structure shift and semantic distortion during generation, and provides reliable augmented samples for subsequent discriminative learning. In summary, $L_{align}$ and $L_{con}$ cooperate to guarantee the semantic reliability of augmented samples, while SDSS and MSSR improve the quality of augmented samples from the perspectives of spatial continuity modeling and semantic diversity generation respectively. All components jointly boost the cross-scene classification performance of MSCGnet.

Furthermore, Table 10, Table 11 and Table 12 compare the performance of different scanning strategies including the proposed SDSS, sequential scanning, snake scanning, diagonal scanning and random scanning. SDSS achieves the highest OA values of 81.62%, 86.81% and 86.47% on three tasks and outperforms all other strategies.

For the small-size water class with only 285 source domain samples in the Houston dataset, SDSS improves the classification accuracy from 83.42% under sequential scanning to 100.00%. For the elongated linear road class in the Houston dataset, SDSS achieves a classification accuracy of 57.38%, ranking first among all scanning strategies. On the YC dataset, the classification accuracy of the river and sea water classes improves by 41.58 and 22.74 percentage points respectively relative to sequential scanning. The three boundary land cover classes of asphalt, bitumen and shadow in the Pavia dataset are easily spectrally confused with adjacent land covers, and SDSS still achieves the optimal classification accuracy for all of them.

However, SDSS delivers a classification accuracy 1 to 4 percentage points lower than several scanning strategies for large-area, spatially continuous and texturally homogeneous land covers, including stressed grass, meadow, bare soil, residential buildings and non-residential buildings. The tokens within patches of these land covers carry highly consistent semantic information, so the performance gain brought by scanning order becomes saturated. For the reed class in the YC dataset, SDSS only achieves a classification accuracy of 20.09%, which is far lower than the 70.90% of sequential scanning and the 72.54% of snake scanning. The reed class contains the fewest source domain samples among all land cover categories, with just 132 samples, and exhibits fragmented and intermingled spatial distribution. This finding demonstrates that the central pixel prior assumption underlying SDSS will be compromised when the central pixel of an image patch corresponds to a mixed impure pixel.

### 4.5. Comparison Experiment

Table 13, Table 14 and Table 15 report the per-category classification accuracy, overall OA and KC of MSCGnet and eight comparison methods on the Houston, Pavia and YC tasks respectively. The best results are marked in bold.

MSCGnet achieves the optimal Overall Accuracy (OA) and Kappa Coefficient (KC) across all three cross-scene classification tasks. Specifically, it obtains an OA of 81.62% and a KC of 67.83% on the Houston task, an OA of 86.81% and a KC of 84.16% on the Pavia task, and an OA of 86.47% and a KC of 82.10% on the YC task. Compared with the second-ranked baseline model on each task, MSCGnet improves OA by 1.92%, 1.36% and 1.64%, respectively.

In terms of per-category classification accuracy, MSCGnet reaches the highest accuracy of 81.45% for the residential building category in the Houston task. This advantage mainly stems from the CAM-based spatial constraint mechanism in the SCGD module, which strengthens the discriminability between man-made ground objects with spatial structural features under cross-scene spectral distribution shifts. Nevertheless, for the categories of healthy grass and stressed grass, MSCGnet only achieves classification accuracies of 62.23% and 73.40%, which are lower than ADnet’s 77.38% and RCRAnet’s 93.72%. This is because these two vegetation categories feature large coverage and uniform textures; algorithms that place more emphasis on adversarial optimization or rank-based feature refinement can achieve better classification performance on such ground objects.

For the Pavia task, MSCGnet attains the highest classification accuracies of 92.81% and 88.61% for the asphalt and meadow categories, respectively. These two ground object types share similar spectral signatures but exhibit distinctly different spatial distribution patterns. The outstanding performance of MSCGnet mainly benefits from the MSSR module, which leverages multi-scale receptive fields to provide differentiated spatial semantic guidance and effectively boost the discriminative capacity for categories with analogous spectra.

On the YC task, MSCGnet achieves a classification accuracy of 68.22% for the river category, showing a noticeable improvement over most comparison methods. This indicates that the SDSS module can effectively maintain the model’s perception of internal spatial continuity within image patches, enabling the model to precisely capture the spatial extension characteristics of linear ground objects such as rivers. However, MSCGnet only yields an accuracy of 20.09% for the reed category, markedly inferior to FDGnet’s 98.77% and most other competing methods. The reed category contains the smallest number of source domain samples among all ground object classes investigated in this paper, with merely 132 samples. Its spectral characteristics are highly similar to those of paddy fields and fallow land, while its spatial distribution consists of scattered patches without continuous extended spatial structures. Under such conditions, the center–periphery spatial prior relied upon by SDSS cannot function adequately, and its advantages are therefore weakened to a certain extent.

**Figure 11:**
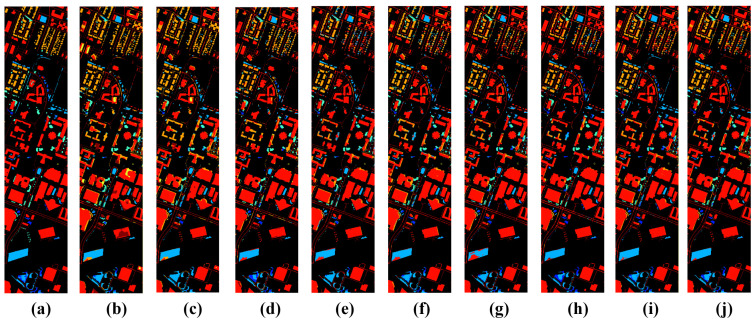
Visual representation of classification results on the Houston task obtained with different methods including: (**a**) ground-truth; (**b**) VREx (70.97%); (**c**) GroupDRO (72.73%); (**d**) LDGnet (79.31%); (**e**) LLURnet (77.58%); (**f**) FDGnet (77.36%); (**g**) ISDGS (76.83%); (**h**) ADnet (79.23%); (**i**) RCRAnet (79.93%); (**j**) ours (82.12%).

**Figure 12:**
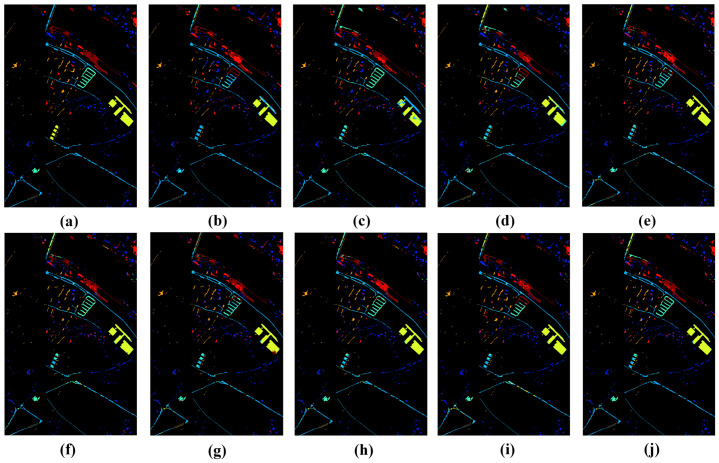
Visual representation of classification results on the Pavia task obtained with different methods including: (**a**) ground-truth; (**b**) VREx (75.29%); (**c**) GroupDRO (75.69%); (**d**) LDGnet (80.24%); (**e**) LLURnet (81.16%); (**f**) FDGnet (83.79%); (**g**) TSDAnet (82.87%); (**h**) ADnet (83.31%); (**i**) RCRAnet (85.79%); (**j**) ours (87.92%).

**Figure 13:**
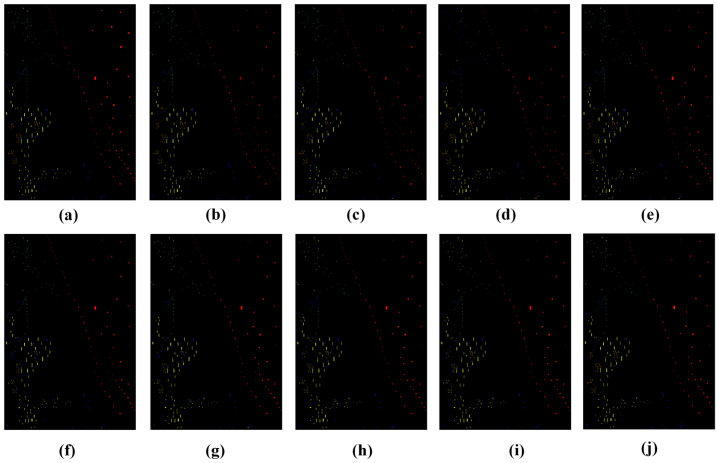
Visual representation of classification results on the YC task obtained with different methods including: (**a**) ground-truth; (**b**) VREx (69.69%); (**c**) GroupDRO (70.36%); (**d**) LDGnet (65.79%); (**e**) LLURnet (83.91%); (**f**) FDGnet (80.37%); (**g**) TSDAnet (79.92%); (**h**) ADnet (84.93%); (**i**) RCRAnet (85.02%); (**j**) ours (87.40%).

Visualization results of classification maps for all methods on three datasets are shown in Figure 11, Figure 12 and Figure 13. Overall, classification maps of MSCGnet are most consistent with ground-truth maps across three tasks, with less pixel confusion at ground object boundaries and stronger spatial coherence of classification regions. In the Houston task, MSCGnet obtains clearer and more accurate classification boundaries for residential buildings in the upper-left area and reduces misclassification of residential buildings as non-residential buildings. In the Pavia task, MSCGnet outperforms other methods in identifying asphalt and meadow. In the YC task, MSCGnet recognizes the slender strip structure of rivers more completely with fewer missed detections and misclassifications, further demonstrating the generalization advantages of MSCGnet for small-sample and fine-grained ground object categories.

Table 16 compares the training time, test time, and model parameters of all methods on the three datasets. The parameter sizes of MSCGnet are only 0.41 MB, 0.50 MB and 0.59 MB on three tasks respectively, which are far smaller than all comparison methods. Meanwhile, MSCGnet achieves the shortest training time and test time. In terms of test efficiency, this superiority comes from the fact that only the lightweight feature extraction backbone and classification head of SCGD are retained for computation during the test phase. The outstanding training efficiency benefits mainly from three factors:

1. MSSG is built on Mamba SSMs with linear computational complexity for sequence modeling, which is more efficient than Transformers with quadratic complexity. It maintains the capability of modeling long-range dependencies while greatly reducing computational overhead.
2. MSSR adopts lightweight multi-scale depthwise separable convolutions, top-k sparse semantic selection and low-rank decomposition of semantic pools. Only a small number of semantic prototypes are activated sparsely, avoiding complex attention calculations and large-scale parameters while enhancing feature representation capability.

3. SCGD generates CAMs by reusing classification head weights without constructing complex domain discriminators or adversarial learning modules, which further reduces model complexity and training cost.

In conclusion, MSCGnet can fully mine spatial semantic information of cross-scene HSI data with few parameters and low computational overhead, achieving an excellent balance between classification accuracy and model efficiency and possessing high practical application value and deployment potential.

**Table 16:** Computational cost on three datasets with different methods.

Model	VREx	GroupDRO	LDGnet	LLURnet	FDGnet	ISDGS	ADnet	RCRAnet	Ours
Dataset	Houston 2018 (Target)								
Train (s)	6.53	5.93	32.85	10.93	6.39	3.86	10.14	30.72	2.29
Test (s)	6.27	5.45	11.23	5.64	3.24	3.51	4.32	18.38	1.49
Params (MB)	10.80	10.80	34.22	0.55	3.14	0.62	1.48	0.90	0.41
Dataset	Pavia Center (Target)								
Train (s)	8.46	7.62	111.92	34.75	19.56	7.08	23.89	66.12	6.01
Test (s)	8.01	7.14	50.42	18.64	10.47	6.47	11.54	35.42	1.80
Params (MB)	10.96	10.96	35.10	0.58	4.04	0.65	2.18	1.88	0.50
Dataset	ZY-YC (Target)								
Train (s)	5.48	4.95	17.94	5.69	4.72	1.86	4.14	10.38	1.25
Test (s)	5.13	4.28	8.56	2.58	2.35	1.43	2.21	6.49	0.66
Params (MB)	11.33	11.33	35.80	0.60	4.93	0.68	2.78	3.01	0.59

### 4.6. T-SNE Visualization Analysis

To conduct a more direct analysis of whether the augmented samples generated by MSSG for domain expansion are semantically valid instead of arbitrary random perturbations, we adopt t-SNE to visualize the deep feature distributions corresponding to source domain samples, MSSG-generated augmented samples for domain expansion, and the discriminator output features of SCGD derived from both groups of samples across three cross-scene tasks.

**Figure 14:**
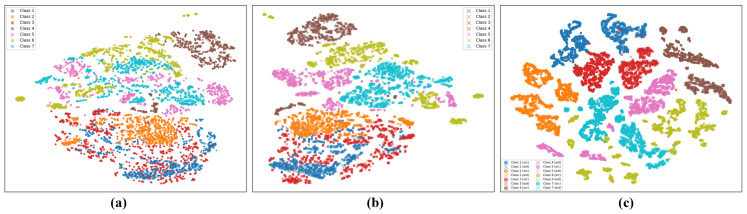
Visualization of source domain and extended domain sample using t-SNE on the Houston task: (**a**) source domain samples; (**b**) extended domain samples; (**c**) features output by the discriminator.

**Figure 15:**
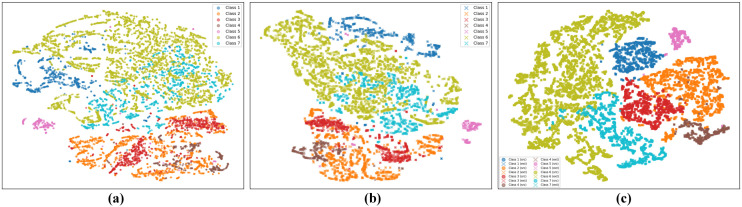
Visualization of source domain and extended domain sample using t-SNE on the Pavia task: (**a**) source domain samples; (**b**) extended domain samples; (**c**) features output by the discriminator.

**Figure 16:**
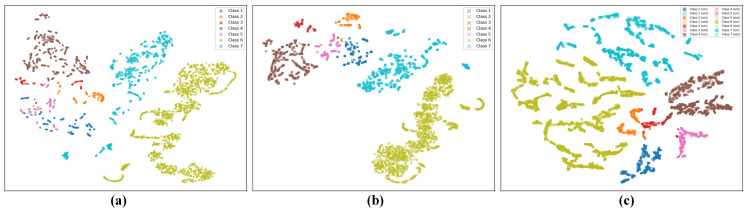
Visualization of source domain and extended domain sample using t-SNE on the YC task: (**a**) source domain samples; (**b**) extended domain samples; (**c**) features output by the discriminator.

As shown in Figure 14, Figure 15 and Figure 16, the extended domain samples in subplot (b) neither collapse onto the source domain distribution in subplot (a) nor scatter randomly across the feature space. Instead, they form a much broader distribution coverage around each class cluster while preserving the inherent class structure of the source domain. This observation indicates that MSSG expands the local feature coverage of each category without destroying intra-class semantic consistency, which is consistent with the design goal of MSSR: generating diverse augmented samples via spatially differentiated semantic prompts. Meanwhile, the CAM alignment loss $L_{align}$ and CAM contrastive loss $L_{con}$ collaboratively confine this expansion within semantically valid boundaries.

Subplot (c) further visualizes features extracted by the SCGD after jointly processing source domain samples and MSSG-generated extended domain samples. For all three tasks, the class clusters in subplot (c) are more compact and exhibit better inter-class separability compared with the raw feature distributions in (a) and (b). This verifies that the discriminative information brought by augmented samples is retained and further enhanced after SCGD processing. The results demonstrate that MSSG-generated augmented samples strike a balance between diversity and semantic validity, and such diversity ultimately evolves into a more discriminative feature space structure.

We also notice varying degrees of cluster expansion across different tasks. The Houston task presents a relatively wider expansion range, which matches its limited source domain sample size—this task benefits the most from the expanded feature coverage brought by sample augmentation. In contrast, the Pavia and YC tasks with abundant source domain samples show milder expansion of augmented sample clusters around their already fully covered class regions.

### 4.7. Failure Case Analysis

**Figure 17:**
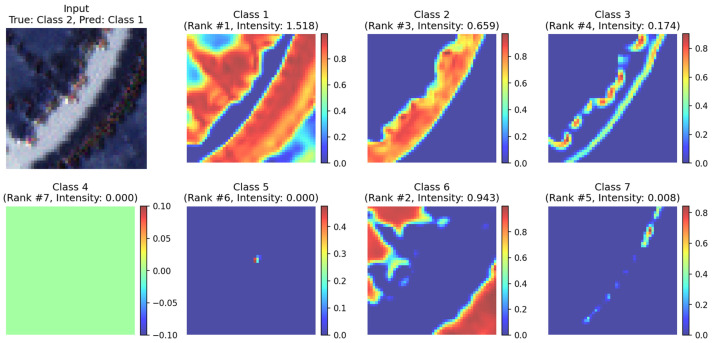
Misclassification case (True: Class 2, Pred: Class 1).

**Figure 18:**
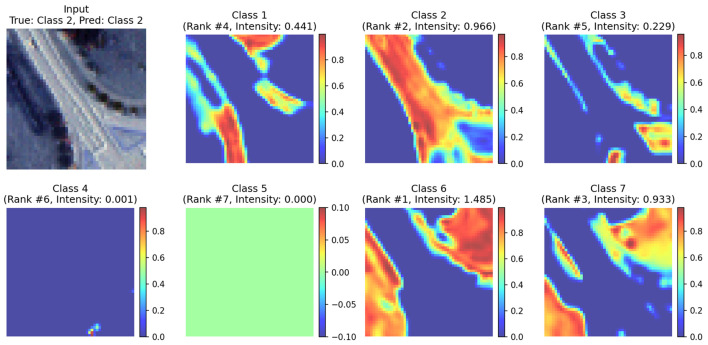
Correct-classification but CAM-misaligned case (True: Class 2, Pred: Class 2).

CAMs inherently deliver coarse-grained spatial responses back-projected from the weights of the classification head. To thoroughly investigate the limitations imposed by CAM supervision, we select two representative failure cases for analysis, corresponding to two scenarios: incorrect category prediction, and correct prediction with spatially inconsistent CAM responses relative to the predicted category, as illustrated in Figure 17 and Figure 18.

The sample visualized in Figure 17 has a ground-truth label of asphalt (Class 2), yet the model misclassifies it as tree (Class 1). The input false-color image depicts a narrow strip-shaped land cover running through the scene center, which matches the typical spatial morphology of asphalt pavement. The CAM corresponding to the ground-truth asphalt exhibits distinct responses along this strip structure, albeit with the third-highest intensity (intensity = 0.659). By contrast, the CAM for the mispredicted tree not only covers the target strip itself but also spreads extensively across the vegetated background flanking the strip, attaining the maximum intensity (intensity = 1.518). The second-ranked meadow (Class 6) CAM responses are entirely confined to diagonal regions irrelevant to the target strip, representing typical background activations. This misclassification therefore does not stem from the model’s inability to perceive road semantics; instead, the strong background vegetation responses of the tree class suppress the genuine asphalt activations over the target region, revealing that the classifier over-relies on co-occurring background cues.

The sample in Figure 18 is also labeled asphalt and receives a correct final prediction, yet the ranking of CAM intensities contradicts the prediction result: the asphalt CAM only ranks second (intensity = 0.966), while the top-ranked meadow CAM (intensity = 1.485) concentrates its high-intensity activations on structures in the upper-right corner of the image that bear no relation to the target road. This demonstrates that, after the classification head aggregates spatial responses globally via global average pooling, the model can still render correct predictions by accumulating discriminative evidence of asphalt across remaining regions, even when a non-target class exhibits substantially stronger local responses. Accordingly, accurate predictions do not guarantee CAM activations that spatially match the semantic scope of target objects, pointing out an easily underappreciated limitation when employing CAMs for spatial supervision.

Collectively, the two aforementioned phenomena reveal that the CAM alignment loss $L_{align}$ embedded within SCGD enforces consistency constraints solely on the CAM channel corresponding to the given ground-truth label, without validating the spatial localization quality of this channel itself. If the source domain classifier learns inaccurate discriminative regions due to spectral–spatial similarities across classes or inherent co-occurrence biases from background elements, $L_{align}$ will further force alignment of these erroneous yet uniform spatial activation patterns between source domain and extended domain samples. This constitutes a potential limitation of the proposed method. For future work, we plan to design an explicit background suppression term to constrain the activations of non-target classes within the spatial scope of target objects, mitigating the adverse impacts of spatial attribution bias from the source domain classifier on cross-domain generalization performance.

## 5. Conclusions

This paper proposes a novel DG method named multi-scale semantic selection and spatial constraint-guided network (MSCGnet) for cross-scene HSI classification. MSCGnet is mainly composed of MSSG and SCGD. With the collaboration of SDSS and MSSR, MSSG maintains the integrity of sample spatial structure, adaptively mines spatial semantic information at different scales, and modulates Mamba SSMs via dynamic semantic prompts to generate extended domain samples with rich semantic diversity. Specifically, SDSS reorganizes tokens in a center-to-periphery diffusion order to enhance the capability of Mamba to model spatial continuity of HSI samples. MSSR constructs dynamic semantic prompts for each token via sparse semantic prototype selection mechanisms and guides the model to generate augmented features with differentiated distributions. On the other hand, SCGD establishes spatial constraint mechanisms using CAMs. Joint optimization of classification loss, CAM alignment loss and CAM contrastive loss constrains the spatial activation distribution of augmented samples, ensuring consistent category semantics and stronger category discriminability. This effectively improves the cross-scene generalization performance of the model. Extensive experiments on three public cross-scene HSI datasets verify that MSCGnet achieves state-of-the-art classification performance with low parameter size and high training efficiency, presenting a superior trade-off between accuracy and efficiency. Future work will be carried out in two directions: first, exploring more refined spatial perception methods to further enhance the diversity and controllability of augmented samples; second, introducing frequency domain disentanglement into the cross-scene HSI classification framework to further improve model robustness and generalization capability for complex unseen target domains.

## Figures and Tables

**Table 1:** Quantity of source and target samples in the Houston dataset.

Class		Number of Samples	
No.	Name	Houston 2013 (Source)	Houston 2018 (Target)
1	Grass Healthy	345	1353
2	Grass Stressed	365	4888
3	Trees	365	2766
4	Water	285	22
5	Residential Buildings	319	5347
6	Non-residential Buildings	408	32,459
7	Road	443	6365
Total		2530	53,200

**Table 2:** Quantity of source and target samples in the Pavia dataset.

Class		Number of Samples	
No.	Name	University of Pavia (Source)	Pavia Center (Target)
1	Tree	3064	7598
2	Asphalt	6631	9248
3	Brick	3682	2685
4	Bitumen	1330	7287
5	Shadow	947	2863
6	Meadow	18,649	3090
7	Bare Soil	5029	6584
Total		39,332	39,335

**Table 3:** Quantity of source and target samples in the YC dataset.

Class		Number of Samples	
No.	Name	GF-YC (Source)	ZY-YC (Target)
1	Architecture	360	451
2	River	217	214
3	Reed	132	244
4	Paddy	832	3026
5	Fallow Land	234	650
6	Sea	2395	2076
7	Offshore Water	1305	1558
Total		5475	8219

**Table 4:** Fine-grained text descriptions of LDGnet on the YC task.

Class Name	Fine-Grained Text
Architecture	Buildings are concentrated in developed regions. Architecture features sharp edges and geometric shapes.
River	Rivers wind through the terrain with clear boundaries. Rivers reflect surrounding vegetation and sky.
Reed	Reeds form thickets along riverbanks. Reeds sway gently in the wind.
Paddy	Paddy fields are neatly divided into plots. Paddy fields shimmer under sunlight when flooded.
Fallow Land	Fallow land lacks vegetation and appears barren. Fallow land may show cracks due to dryness.
Sea	Sea extends beyond visible horizons with vast openness. Sea waves create dynamic patterns on the surface.
Offshore Water	Offshore water merges seamlessly with the horizon. Offshore water appears deeper and more mysterious.

**Table 5:** Parameter tuning of patch size on three tasks.

Task	Patch Size				
	9 × 9	11 × 11	13 × 13	15 × 15	17 × 17
Houston	80.41	81.03	81.62	80.38	78.70
Pavia	83.19	83.94	86.81	83.62	82.93
YC	86.38	86.47	85.78	85.08	83.99

**Table 6:** Parameter tuning of semantic prototype count *T* on three tasks.

Task	Semantic Prototype Count *T*				
	8	16	32	64	128
Houston	78.61	80.24	81.62	80.10	79.85
Pavia	82.72	84.40	86.81	85.22	84.97
YC	83.15	84.63	86.47	84.98	84.30

**Table 7:** Parameter tuning of top-*k* sparsity *k* on three tasks.

Task	Top-*k* Sparsity Coefficient *k*				
	1	2	3	4	5
Houston	74.34	78.87	81.62	79.35	78.92
Pavia	80.58	85.12	86.81	83.40	82.71
YC	81.02	83.10	86.47	82.05	81.44

**Table 8:** Comparison between theoretically derived weights and grid search optimal weights on three tasks.

Task	Grid Search Optimal (*λ*_1_, *λ*_2_)	Theoretically Derived (*λ*_1_, *λ*_2_)	Relative Deviation of (*λ*_1_, *λ*_2_)
Houston	(1.00, 1.00)	(0.92, 0.88)	(8.00%, 12.00%)
Pavia	(0.25, 0.50)	(0.22, 0.56)	(12.00%, 12.00%)
YC	(0.25, 0.50)	(0.27, 0.43)	(8.00%, 14.00%)

**Table 9:** Accuracy of ablation experiments on three tasks.

SDSS	MSSR	$L_{align}$	$L_{con}$	Houston		Pavia		YC	
				OA	KC	OA	KC	OA	KC
√		√	√	74.89 ± 1.32	58.13 ± 1.48	82.47 ± 1.89	79.03 ± 1.47	82.12 ± 1.88	76.59 ± 1.75
	√	√	√	80.01 ± 1.54	64.78 ± 1.73	82.11 ± 1.46	78.54 ± 1.34	80.74 ± 1.28	74.42 ± 1.03
√	√	√		77.52 ± 0.93	62.86 ± 1.12	84.68 ± 1.77	80.51 ± 1.87	86.14 ± 1.14	81.57 ± 1.25
√	√		√	73.68 ± 1.24	58.12 ± 1.54	75.93 ± 1.85	70.59 ± 1.46	81.96 ± 1.33	75.69 ± 1.41
√	√	√	√	81.62 ± 0.83	67.83 ± 1.78	86.81 ± 1.43	84.16 ± 1.63	86.47 ± 0.84	82.10 ± 1.39

**Table 10:** Performance of MSCGnet on the Houston task under different scan strategies.

Class	Sequential	Snake	Diagonal	Random	SDSS
1	45.37	50.03	52.26	46.55	62.23
2	81.69	73.53	71.38	71.54	73.40
3	53.40	55.46	55.71	55.24	59.94
4	83.42	85.52	86.34	89.42	100.00
5	77.58	84.10	83.79	84.22	81.45
6	87.32	88.63	92.97	93.11	91.52
7	51.57	52.76	46.74	46.83	57.38
OA (%)	79.15 ± 1.45	80.11 ± 1.63	80.69 ± 1.84	80.83 ± 1.53	81.62 ± 0.83
Kappa (%)	65.45 ± 1.82	66.84 ± 1.25	66.28 ± 1.05	66.50 ± 1.12	67.83 ± 1.78

**Table 11:** Performance of MSCGnet on the Pavia task under different scan strategies.

Class	Sequential	Snake	Diagonal	Random	SDSS
1	79.07	88.64	88.68	81.44	86.19
2	89.67	83.94	89.90	88.45	92.81
3	84.43	78.77	77.43	78.99	83.80
4	79.68	70.45	75.31	80.29	84.81
5	83.76	80.23	84.44	83.59	84.74
6	89.29	87.61	85.63	90.00	88.61
7	69.23	87.97	76.03	77.75	82.61
OA (%)	81.75 ± 1.28	82.69 ± 1.63	83.35 ± 1.14	83.13 ± 1.89	86.81 ± 1.43
Kappa (%)	78.23 ± 1.79	79.32 ± 1.78	80.03 ± 1.23	79.86 ± 1.45	84.16 ± 1.63

**Table 12:** Performance of MSCGnet on the YC task under different scan strategies.

Class	Sequential	Snake	Diagonal	Random	SDSS
1	90.91	97.78	79.60	95.12	99.11
2	26.64	17.76	78.50	35.05	68.22
3	70.90	72.54	0.00	48.36	20.09
4	100.00	100.00	100.00	100.00	100.00
5	79.23	79.23	82.46	85.23	79.54
6	42.87	43.69	55.83	52.26	65.61
7	100.00	100.00	100.00	100.00	100.00
OA (%)	80.65 ± 1.63	81.05 ± 1.53	82.80 ± 1.24	83.28 ± 1.58	86.47 ± 0.84
Kappa (%)	74.54 ± 1.22	75.04 ± 1.68	77.12 ± 1.39	77.93 ± 1.96	82.10 ± 1.39

**Table 13:** Comparison of quantitative classification results of different methods on the Houston task (target: Houston 2018).

Class	VREx	GroupDRO	LDGnet	LLURnet	FDGnet	ISDGS	ADnet	RCRAnet	Ours
1	20.77	15.45	54.99	24.83	55.8	51.59	77.38	57.73	62.23
2	76.90	80.26	72.20	75.16	80.38	69.21	77.19	93.72	73.40
3	53.29	29.07	57.01	58.82	62.15	57.41	58.75	42.91	59.94
4	90.91	100.00	81.82	100.00	100.00	100. 00	90.91	100.00	100.00
5	72.69	75.96	76.77	58.89	76.19	66.41	67.78	79.71	81.45
6	72.42	78.96	89.91	88.43	83.55	89.60	94.43	89.08	91.52
7	59.12	46.74	45.25	58.51	54.83	37.91	20.47	41.82	57.38
OA (%)	70.82 ± 1.29	72.14 ± 1.65	79.02 ± 1.58	77.51 ± 1.53	77.27 ± 0.96	76.58 ± 1.29	79.03 ± 1.38	79.70 ± 1.38	81.62 ± 0.83
Kappa (%)	55.94 ± 1.83	56.79 ± 1.48	63.92 ± 1.97	61.07 ± 1.75	62.47 ± 1.30	58.72 ± 1.68	61.62 ± 1.72	65.20 ± 1.72	67.83 ± 1.78

**Table 14:** Comparison of quantitative classification results of different methods on the Pavia task (target: Pavia Center).

Class	VREx	GroupDRO	LDGnet	LLURnet	FDGnet	ISDGS	ADnet	RCRAnet	Ours
1	76.36	76.01	98.26	87.22	83.64	87.80	92.16	95.05	86.19
2	86.63	83.23	86.65	85.26	79.90	85.93	85.68	82.40	92.81
3	15.49	97.43	48.23	53.33	89.72	67.30	82.16	60.71	83.80
4	73.24	58.14	73.82	87.36	85.10	83.44	87.26	84.41	84.81
5	67.83	73.66	93.96	85.30	91.76	90.92	95.32	98.95	84.74
6	83.06	74.53	53.62	78.90	73.30	72.65	66.31	75.08	88.61
7	63.79	66.25	76.55	71.55	85.84	86.32	67.94	88.90	82.61
OA (%)	74.23 ± 1.33	75.18 ± 1.27	80.14 ± 1.52	81.06 ± 1.68	83.59 ± 1.42	83.94 ± 1.39	83.20 ± 1.35	85.45 ± 1.65	86.81 ± 1.43
Kappa (%)	70.11 ± 1.59	71.08 ± 1.36	76.00 ± 1.72	77.21 ± 1.92	80.41 ± 1.53	80.68 ± 1.46	79.83 ± 1.51	82.47 ± 2.04	84.16 ± 1.63

**Table 15:** Comparison of quantitative classification results of different methods on the YC task (target: ZY-YC).

Class	VREx	GroupDRO	LDGnet	LLURnet	FDGnet	ISDGS	ADnet	RCRAnet	Ours
1	90.02	95.34	55.43	67.18	93.79	91.57	98. 00	99.78	99.11
2	27.57	45.33	0.00	36.92	36.92	25.70	8.41	32.24	68.22
3	60.25	71.31	0.00	54.10	98.77	93.85	77.46	69.26	20.09
4	100.00	100.00	100.00	100.00	98.28	100. 00	100.00	100.00	100.00
5	81.85	76.62	85.69	92.92	60.92	46.31	62.46	51.69	79.54
6	0.00	0.00	0.00	56.70	43.83	64.69	64.21	65.61	65.61
7	100.00	100.00	100.00	100.00	100.00	100.00	100.00	100.00	100.00
OA (%)	69.40 ± 1.78	70.18 ± 1.37	65.59 ± 1.32	83.70 ± 1.18	80.07 ± 1.30	84.26 ± 1.35	84.83 ± 1.21	84.80 ± 0.79	86.47 ± 0.84
Kappa (%)	54.71 ± 1.79	61.36 ± 1.54	54.71 ± 1.62	78.47 ± 1.32	73.96 ± 1.67	79.30 ± 1.79	79.95 ± 1.39	79.85 ± 1.03	82.10 ± 1.39

## Data Availability

Data are contained within the article.
